# Variational Information Bottleneck for Semi-Supervised Classification

**DOI:** 10.3390/e22090943

**Published:** 2020-08-27

**Authors:** Slava Voloshynovskiy, Olga Taran, Mouad Kondah, Taras Holotyak, Danilo Rezende

**Affiliations:** 1 Department of Computer Science, University of Geneva, 1227 Carouge, Switzerland; olga.taran@unige.ch (O.T.); mouad.kondah@etu.unige.ch (M.K.); taras.holotyak@unige.ch (T.H.); 2 DeepMind, London N1C 4AG, UK; danilor@google.com

**Keywords:** information bottleneck principle, deep networks, semi-supervised classification, latent space representation, hand crafted priors, learnable priors, regularization

## Abstract

In this paper, we consider an information bottleneck (IB) framework for semi-supervised classification with several families of priors on latent space representation. We apply a variational decomposition of mutual information terms of IB. Using this decomposition we perform an analysis of several regularizers and practically demonstrate an impact of different components of variational model on the classification accuracy. We propose a new formulation of semi-supervised IB with hand crafted and learnable priors and link it to the previous methods such as semi-supervised versions of VAE (M1 + M2), AAE, CatGAN, etc. We show that the resulting model allows better understand the role of various previously proposed regularizers in semi-supervised classification task in the light of IB framework. The proposed IB semi-supervised model with hand-crafted and learnable priors is experimentally validated on MNIST under different amount of labeled data.

## Notations

We will denote a joint generative distribution as $p_{\theta }\left(x,z\right)=p_{\theta }\left(z\right)p_{\theta }\left(x|z\right)$, whereas marginal $p_{\theta }\left(z\right)$ is interpreted as a targeted distribution of latent space and marginal $p_{\theta }\left(x\right)=E_{p_{\theta }\left(z\right)}\left[p_{\theta }\left(x|z\right)\right]=\int_{z}p_{\theta }\left(x|z\right)p_{\theta }\left(z\right)dz$ as a generated data distribution with a generative model described by $p_{\theta }\left(x|z\right)$, where E stands for the expected value. A joint data distribution $q_{\phi }\left(x,z\right)=p_{D}\left(x\right)q_{\phi }\left(z|x\right)$, where $p_{D}\left(x\right)$ denotes an empirical data distribution and $q_{\phi }\left(z|x\right)$ is an inference or encoding model and marginal $q_{\phi }\left(z\right)$ denotes a “true” or “aggregated” distribution of latent space data. We will denote parameters of encoders as $\phi_{a}$ and $\phi_{z}$, and those of decoders as $\theta_{c}$ and $\theta_{x}$. The discriminators corresponding to Kullback–Leibler divergences are denoted as $D_{x}$ where the subscript indicates the space to which this discriminator is applied to. The cross-entropy metrics are denoted as $D_{x\hat{x}}$, where the subscript indicates the corresponding vectors. X denotes random vector, while the corresponding realization is denoted as x.

## 1. Introduction

The deep supervised classifiers demonstrate an impressive performance when the amount of labeled data is large. However, their performance significantly deteriorates with the decrease of labeled samples. Recently, semi-supervised classifiers based on deep generative models such as VAE (M1 + M2) [1], AAE [2], CatGAN [3], etc., along with several other approaches based on multi-view and contrastive metrics just to mention the most recent ones [4,5], are considered to be a solution to the above problem. Besides the remarkable reported results, the information theoretic analysis of semi-supervised classifiers based on generative models and the role of different priors aiming to fulfil the gap in the lack of labeled data remain little studied. Therefore, in this paper we will try to address these issues using IB principle [6] and practically compare different priors on the same architecture of classifier.

Instead of considering the latent space of generative models such as VAE (M1 + M2) [1] and AAE [2] trained in the unsupervised way as suitable features for the classification, we will depart from the IB formulation of supervised classification, where we consider an encoder-decoder formulation of classifier and impose priors on its latent space. Thus, we study an approach to semi-supervised classification based on an IB formulation with a variational decomposition of IB compression and classification mutual information terms. To deeper understand the role and impact of different elements of variational IB on the classification accuracy, we consider two types of priors on the latent space of classifier: (i) hand-crafted and (ii) learnable priors. *Hand-crafted* latent space priors impose constraints on a distribution of latent space by fitting it to some targeted distribution according to the variational decomposition of the compression term of the IB. This type of latent space priors is well known as an information dropout [7]. One can also apply the same variational decomposition to the classification term of the IB, where the distribution of labels is supposed to follow some targeted class distribution to maximize the mutual information between inferred labels and targeted ones. This type of class label space regularization reflects an adversarial classification used in AAE [2] and CatGAN [3]. In contrast, *learnable* latent space priors aim at minimizing the need in human expertise in imposing priors on the latent space. Instead, the learnable priors are learned directly from unlabeled data using auto-encoding (AE) principle. In this way, the learnable priors are supposed to compensate the lack of labeled data in the semi-supervised learning yet minimizing the need in the hand-crafted control of the latent space distribution.

We demonstrate that several state-of-the-art models such as AAE [2], CatGAN [3], VAE (M1 + M2) [1], etc., can be considered to be instances of the variational IB with the learnable priors. At the same time, the role of different regularizers in the hand-crafted semi-supervised learning is generalized and linked to known frameworks such as information dropout [7].

We evaluate our model using standard dataset MNIST on both hand-crafted and learnable features. Besides revealing the impact of different components of variational IB factorization, we demonstrate that the proposed model outperforms prior works on this dataset.

Our main contribution is three-fold: (i) We propose a new formulation of IB for the semi-supervised classification and use a variational decomposition to convert it into a practically tractable setup with learnable parameters. (ii) We develop the variational IB for two classes of hand-crafted and learnable priors on the latent space of classifier and show its link to the state-of-the-art semi-supervised methods. (iii) We investigate the role of these priors and different regularizers in the classification, latent and reconstruction spaces for the same fixed architecture under the different amount of training data.

## 2. Related Work

**Regularization techniques in semi-supervised learning**: Semi-supervised learning tries to find a way to benefit from a large number of unlabeled samples available for training. The most common way to leverage unlabeled data is to add a special regularization term or some mechanism to better generalize to unseen data. The recent work [8] identifies three ways to construct such a regularization: (i) entropy minimization, (ii) consistency regularization and (iii) generic regularization. The entropy minimization [9,10] encourages the model to output confident predictions on unlabeled data. In addition, more recent work [3] extends this concept to adversarially generated samples or fakes for which the entropy of class label distribution was suggested to be maximized. Finally, the adversarial regularization of label space was considered in [2], where the discriminator was trained to ensure the labels produced by the classifier follow a prior distribution, which was defined to be a categorical one. The consistency regularization [11,12] encourages the model to produce the same output distribution when its inputs are perturbed. Finally, the generic regularization encourages the model to generalize well and avoid overfitting the training data. It can be achieved by imposing regularizers and corresponding priors on the model parameters or feature vectors.

In this work, we implicitly use the concepts of all three forms of considered regularization frameworks. However, instead of adding additional regularizers to the baseline classifier as suggested by the framework in [8], we will try to derive the corresponding counterparts from a semi-supervised IB framework. In this way, we will try to justify their origin and investigate their impact on overall classification accuracy for the same system architecture.

**Information bottleneck:** In the recent years, the IB framework [6] is considered to be a theoretical framework for analysis and explanation of supervised deep learning systems. However, as shown in [13], the original IB framework faces several practical issues: (i) for the deterministic deep networks, either the IB functional is infinite for network parameters, that leads to the ill-posed optimization problem, or it is piecewise constant, hence not admitting gradient-based optimization methods, and (ii) the invariance of the IB functional under bijections prevents it from capturing properties of the learned representation that are desirable for classification. In the same work, the authors demonstrate that these issues can be partly resolved for stochastic deep networks, networks that include a (hard or soft) decision rule, or by replacing the IB functional with related, but more well-behaved cost functions. It is important to mention that the same authors also note that rather than trying to repair the inherent problems in the IB functional, a better approach may be to design regularizers on latent representation enforcing the desired properties directly.

In our work, we extend these ideas using variational approximation approach suggested in [14] and that was also applied to unsupervised models in the previous work [15,16]. More particularly, we extend the IB framework to the semi-supervised classification and as discussed above we will consider two different ways of regularization of the latent space of classifier, i.e., either using traditional hand-crafted priors or suggested learnable priors. Although we do not consider the semi-supervised clustering and conditional generation in this work, the proposed findings can be extended to these problems in a way similar to prior works such as AAE [2], ADGM [17] and SeGMA [18].

**The closest works:** The proposed framework is closely related to several families of semi-supervised classifiers based on generative models. VAE (M1 + M2) [1] combines latent-feature discriminative model M1 and generative semi-supervised model M2. A new latent representation is learned using the generative model from M1 and subsequently a generative semi-supervised model M2 is trained using embeddings from the first latent representation instead of the raw data. Semi-supervised AAE classifier [2] is based on the AE architecture, where the encoder of AE outputs two latent representations: one representing class and another style. The latent class representation is regularized by an adversarial loss forcing it to follow categorical distribution. It is claimed that it plays an essential role for the overall classification performance. The latent style representation is regularized to follow Gaussian distribution. In both cases of VAE and AAE, the mean square error (MSE) metric is used for the reconstruction space loss. CatGAN [3] is an extension of GAN and is based on an objective function that trades-off mutual information between observed examples and their predicted categorical class distribution, against robustness of the classifier to an adversarial generative model.

**Figure 1:**
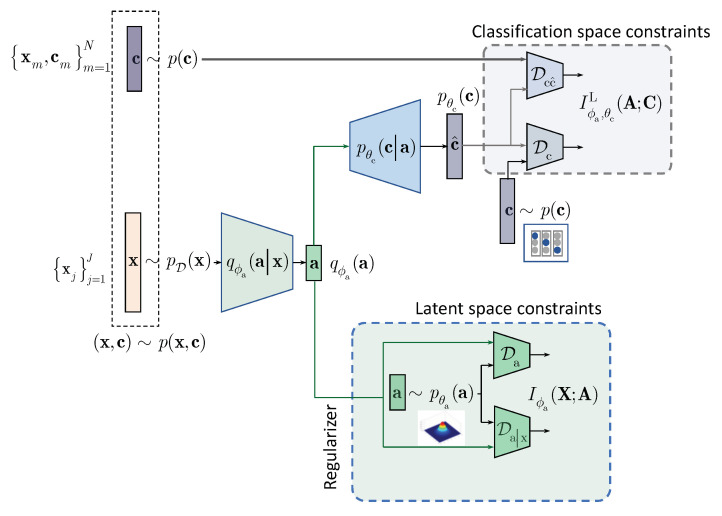
Classification with the hand-crafted latent space regularization.

**Figure 2:**
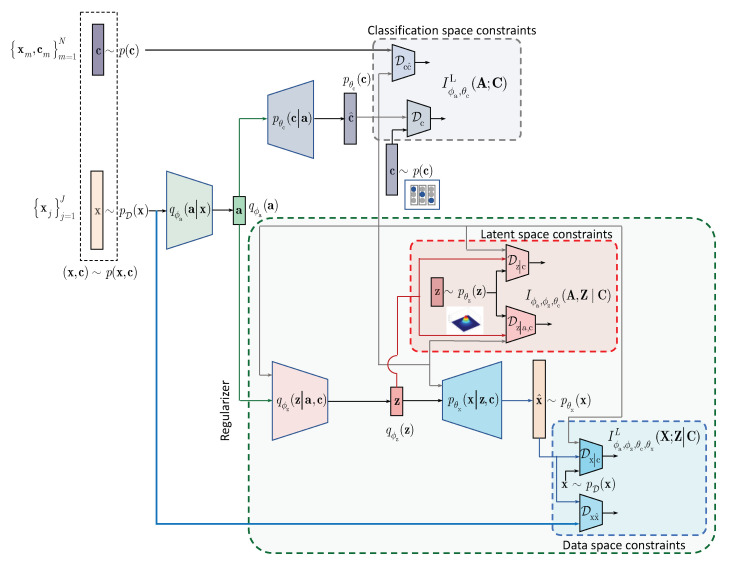
Classification with the learnable latent space regularization.

In contrast to the above approaches and following the IB framework, we formulate the semi-supervised classification problem as a training of classifier that aims at compressing the input x to some latent data a via an encoding that is supposed to retain only class relevant information that is controlled by a decoder as shown in Figure 1. If the amount of labeled data is sufficiently large, the supervised classifier can achieve this goal. However, when the amount of labeled examples is small such an encoder-decoder pair representing an IB-driven classifier is regularized by a latent space and adversarial label space regularizers to fill the gap in training data. The adversarial label space regularization was already used in AAE and CatGAN. The latent space regularization in the scope of IB framework was reported in [7]. In this paper, we demonstrate that both label and latent space regularizations are instances of the generalized IB formulation developed in Section 3. At the same time, in contrast to the hypothesis that the considered label space and latent space regularizations are the driving factors behind the success of semi-supervised classifiers, we demonstrate that the hand-crafted priors considered in these models cannot completely fulfil the lack of labelled data and lead to relatively poor performance in comparison to a fully supervised system based on a sole cross-entropy metric. For these reasons, we analyze another mechanism of regularization of latent space based on learnable priors as shown in Figure 2 and developed in Section 4. Along this line, we provide an IB formulation of AAE and explain the driving mechanisms behind its success as an instance of IB with learnable priors. Finally, we present several extensions that explain the IB origin and role of adversarial regularization in the reconstruction space.

**Summary:** The considered methods of semi-supervised learning can be differentiated based on: (i) *the targeted tasks* (auto-encoding, clustering, generation or classification that can be accomplished depending on available labeled data); (ii) *the architecture in terms of the latent space representation* (with a single representation vector or with multiple representation vectors); (iii) *the usage of IB or other underlying frameworks* (methods derived from the IB directly or using regularization techniques); (iv) *the label space regularization* (based on available unlabeled data, augmented labeled data, synthetically generated labeled and unlabeled data, especially designed adversarial examples); (v) *the latent space regularization* (hand-crafted regularizers and priors or learnable priors under the reconstruction and constrastive setups) and (vi) *the reconstruction space regularization in case of reconstruction setup* (based on unlabeled and labeled data, augmented data under certain perturbations, synthetically generated examples).

In this work, our main focus is the latent space regularization for the hand-crafted and learnable priors under the reconstruction setup within the IB framework. Our main task is the semi-supervised classification. We will not consider any augmentation and adversarial techniques besides a simple stochastic encoding based on the addition of data independent noise at the system input or even deterministic encoding without any form of augmentation. The regularization of the label space and reconstruction space is solely based on the terms derived from the IB framework and only includes available labeled and unlabeled data without any form of augmentation. In this way, we want to investigate the role and impact of the latent space regularization as such in the IB-based semi-supervised classification. The usage of the above mentioned techniques of augmentation should be further investigated and will likely provide an additional performance improvement.

## 3. IB with Hand-Crafted Priors (HCP)

We assume that a semi-supervised classifier has an access to ${\left\{x_{m},c_{m}\right\}}_{m=1}^{N}$ training labeled samples, where $x_{m}\in R^{D}$ denotes $m^{th}$ data sample and $c_{m}$ corresponding encoded class label from the set $\left\{1,2,\cdots ,M_{c}\right\}$, generated from the joint distribution p(c,x), and non-labeled data samples ${\left\{x_{j}\right\}}_{j=1}^{J}$ with J≫N. To integrate the knowledge about the labeled and non-labeled data at training, one can formulate the IB as:

(1) $$L^{\mathrm{HCP}}\left(\phi_{a}\right)=I_{\phi_{a}}\left(X;A\right)-\beta_{c}I_{\phi_{a}}\left(A;C\right),$$

where a denotes the latent representation, $\beta_{c}$ is a Lagrangian multiplier and the IB terms are defined as $I_{\phi_{a}}\left(X;A\right)=E_{q_{\phi_{a}}\left(x,a\right)}\left[\log\frac{q_{\phi_{a}}\left(a|x\right)}{q_{\phi_{a}}\left(a\right)}\right]$ and $I_{\phi_{a}}\left(A;C\right)=E_{p(c,x)}\left[E_{q_{\phi_{a}}\left(a|x\right)}\left[\log\frac{q_{\phi_{a}}\left(c|a\right)}{p(c)}\right]\right]$.

According to the above IB formulation the encoder $q_{\phi_{a}}\left(a|x\right)$ is trained to minimize the mutual information between X and A while ensuring that the decoder $q_{\phi_{a}}\left(c|a\right)$ can reliably decide on labels C from the compressed representation A. The trade-off between the compression and recognition terms is controlled by $\beta_{c}$. Thus, it is assumed that the information retained in the latent representation A represents the sufficient statistics for the class labels C.

However, since optimal $q_{\phi_{a}}\left(c|a\right)$ is unknown, the second term $I_{\phi_{a}}\left(A;C\right)$ is lower bounded by $I_{\phi_{a},\theta_{c}}\left(A;C\right)$ using a variational approximation $p_{\theta_{c}}\left(c|a\right)$:

(2) $$\begin{array}{cc} I_{\phi_{a}}\left(A;C\right) & ≜E_{p(c,x)}\left[E_{q_{\phi_{a}}\left(a|x\right)}\left[\log\frac{q_{\phi_{a}}\left(c|a\right)}{p(c)}\right]\right]=E_{p(c,x)}\left[E_{q_{\phi_{a}}\left(a|x\right)}\left[\log\frac{q_{\phi_{a}}\left(c|a\right)}{p(c)}\frac{p_{\theta_{c}}\left(c|a\right)}{p_{\theta_{c}}\left(c|a\right)}\right]\right]\\ & =E_{p(c,x)}\left[E_{q_{\phi_{a}}\left(a|x\right)}\left[\log\frac{p_{\theta_{c}}\left(c|a\right)}{p(c)}\right]\right]+E_{p(c,x)}\left[E_{q_{\phi_{a}}\left(a|x\right)}\left[\log\frac{q_{\phi_{a}}\left(c|a\right)}{p_{\theta_{c}}\left(c|a\right)}\right]\right]\\ & =E_{p(c,x)}\left[E_{q_{\phi_{a}}\left(a|x\right)}\left[\log\frac{p_{\theta_{c}}\left(c|a\right)}{p(c)}\right]\right]+E_{p(c,x)}\left[D_{\mathrm{KL}}(q_{\phi_{a}}\left(c|a\right)\left|\right|p_{\theta_{c}}\left(c|a\right))\right]\\ & \geq E_{p(c,x)}\left[E_{q_{\phi_{a}}\left(a|x\right)}\left[\log\frac{p_{\theta_{c}}\left(c|a\right)}{p(c)}\right]\right], \end{array}$$

where $D_{\mathrm{KL}}(q_{\phi_{a}}\left(c|a\right)\left|\right|p_{\theta_{c}}\left(c|a\right))=E_{q_{\phi_{a}}\left(a|x\right)}\left[\log\frac{q_{\phi_{a}}\left(c|a\right)}{p_{\theta_{c}}\left(c|a\right)}\right]$ and the inequality follows from the fact that $D_{\mathrm{KL}}(q_{\phi_{a}}\left(c|a\right)\left|\right|p_{\theta_{c}}\left(c|a\right))\geq 0$. We denote the term $I_{\phi_{a},\theta_{c}}\left(A;C\right)=E_{p(c,x)}\left[E_{q_{\phi_{a}}\left(a|x\right)}\left[\log\frac{p_{\theta_{c}}\left(c|a\right)}{p(c)}\right]\right]$. Thus, $I_{\phi_{a}}\left(A;C\right)\geq I_{\phi_{a},\theta_{c}}\left(A;C\right)$.

Thus, the IB (1) can be reformulated as:

(3) $$L^{{\mathrm{HCP}}_{L}}\left(\phi_{a},\theta_{c}\right)=I_{\phi_{a}}\left(X;A\right)-\beta_{c}I_{\phi_{a},\theta_{c}}\left(A;C\right).$$

The considered IB is schematically shown in Figure 1 and we will proceed next with the detailed development of each component of the IB formulation.

### 3.1. Decomposition of the First Term: Hand-Crafted Regularization

The first mutual information term $I_{\phi_{a}}\left(X;A\right)$ in (3) can be decomposed using a factorization by a parametric marginal distribution $p_{\theta_{a}}\left(a\right)$ that represents a prior on the latent representation a:

(4) $$\begin{array}{cc} I_{\phi_{a}}\left(X;A\right) & =E_{q_{\phi_{a}}\left(x,a\right)}\left[\log\frac{q_{\phi_{a}}\left(x,a\right)}{q_{\phi_{a}}\left(a\right)p_{D}\left(x\right)}\right]=E_{q_{\phi_{a}}\left(x,a\right)}\left[\log\frac{q_{\phi_{a}}\left(a|x\right)}{q_{\phi_{a}}\left(a\right)}\frac{p_{\theta_{a}}\left(a\right)}{p_{\theta_{a}}\left(a\right)}\right]\\ & =E_{p_{D}\left(x\right)}\underset{D_{a|x}}{\underset{︸}{\left[D_{\mathrm{KL}}\left(q_{\phi_{a}}\left(a|X=x\right)∥p_{\theta_{a}}\left(a\right)\right)\right]}}-\underset{D_{a}}{\underset{︸}{D_{\mathrm{KL}}\left(q_{\phi_{a}}\left(a\right)∥p_{\theta_{a}}\left(a\right)\right)}}\;, \end{array}$$

where the first term denotes the KL-divergence $D_{a|x}≜D_{\mathrm{KL}}\left(q_{\phi_{a}}\left(a|X=x\right)∥p_{\theta_{a}}\left(a\right)\right)=E_{q_{\phi_{a}}\left(a|x\right)}\left[\log\frac{q_{\phi_{a}}\left(a|x\right)}{p_{\theta_{a}}\left(a\right)}\right]$ and the term denotes the KL-divergence $D_{a}≜D_{\mathrm{KL}}\left(q_{\phi_{a}}\left(a\right)∥p_{\theta_{a}}\left(a\right)\right)=E_{q_{\phi_{a}}\left(a\right)}\left[\log\frac{q_{\phi_{a}}\left(a\right)}{p_{\theta_{a}}\left(a\right)}\right]$.

It should be pointed out that the encoding $q_{\phi_{a}}\left(a|x\right)$ can be both stochastic or deterministic. *Stochastic encoding* $q_{\phi_{a}}\left(a|x\right)$ can be implemented via: (a) *multiplicative encoding* applied to the input x as $a=f_{\phi_{a}}\left(x⊙\epsilon \right)$ or in the latent space $a=f_{\phi_{a}}\left(x\right)⊙\epsilon$, where $f_{\phi_{a}}\left(x\right)$ is the output of the encoder, ⊙ denotes the element-wise product and ϵ follows some data independent or data dependent distribution as in information dropout [7]; (b) *additive encoding* applied to the input x as $a=f_{\phi_{a}}\left(x+\epsilon \right)$ with the data independent perturbations, e.g., such as in PixelGAN [19], or in the latent space with generally data-dependent perturbations of form $a=f_{\phi_{a}}\left(x\right)+\sigma_{\phi_{a}}\left(x\right)⊙\epsilon$, where $f_{\phi_{a}}\left(x\right)$ and $\sigma_{\phi_{a}}\left(x\right)$ are outputs of the encoder and ϵ is assumed to be a zero mean unit variance vector such as in VAE [1] or (c) *concatenative/mixing encoding* $a=f_{\phi_{a}}\left(\left[x,\epsilon \right]\right)$ that is generally applied at the input of encoder. Deterministic encoding is based on the mapping $a=f_{\phi_{a}}\left(x\right)$, i.e., no randomization is introduced, e.g., such as one of encoding modalities of AAE [2].

### 3.2. Decomposition of the Second Term

In this section, we factorize the second term in (3) to address the semi-supervised training, i.e., to integrate the knowledge of both non-labeled and labeled data available at training:

(5) $$\begin{array}{cc} I_{\phi_{a},\theta_{c}}\left(A;C\right) & ≜E_{p(c,x)}\left[E_{q_{\phi_{a}\left(a|x\right)}}\left[\log\frac{p_{\theta_{c}}\left(c|a\right)}{p(c)}\frac{p_{\theta_{c}}\left(c\right)}{p_{\theta_{c}}\left(c\right)}\right]\right]\\ & =-E_{p(c)}\left[\log p_{\theta_{c}}\left(c\right)\right]-E_{p(c)}\left[\log\frac{p(c)}{p_{\theta_{c}}\left(c\right)}\right]+E_{p(c,x)}\left[E_{q_{\phi_{a}}\left(a|x\right)}\left[\log p_{\theta_{c}}\left(c|a\right)\right]\right]\\ & =H\left(p\left(c\right);p_{\theta_{c}}\left(c\right)\right)-D_{\mathrm{KL}}\left(p\left(c\right)∥p_{\theta_{c}}\left(c\right)\right)-H_{\theta_{c},\phi_{a}}\left(C|A\right), \end{array}$$

with $H\left(p\left(c\right);p_{\theta }\left(c\right)\right)=-E_{p(c)}\left[\log p_{\theta_{c}}\left(c\right)\right]$ denoting a cross-entropy between p(c) and $p_{\theta_{c}}\left(c\right)$, and $D_{c}≜D_{\mathrm{KL}}\left(p\left(c\right)∥p_{\theta_{c}}\left(c\right)\right)=E_{p(c)}\left[\log\frac{p(c)}{p_{\theta_{c}}\left(c\right)}\right]$ to be a KL-divergence between the prior class label distribution p(c) and the estimated one $p_{\theta_{c}}\left(c\right)$. One can assume different forms of labels’ c encoding but one of the most often used forms is one-hot-label encoding that leads to the categorical distribution p(c)=cat(c).

Finally, the conditional entropy is defined as $D_{c\hat{c}}≜H_{\theta_{c},\phi_{a}}\left(C|A\right)=-E_{p(c,x)}\left[E_{q_{\phi_{a}}\left(a|x\right)}\left[\log p_{\theta_{c}}\left(c|a\right)\right]\right]$.

Since $H(p\left(c\right);p_{\theta_{c}}\left(c\right))\geq 0$, one can lower bound (5) as $I_{\phi_{a},\theta_{c}}\left(A;C\right)\geq I_{\phi_{a},\theta_{c}}^{L}\left(A;C\right)$ where:

(6) $$\begin{array}{c} I_{\phi_{a},\theta_{c}}^{L}\left(A;C\right)≜-\underset{D_{c}}{\underset{︸}{D_{\mathrm{KL}}\left(p\left(c\right)∥p_{\theta_{c}}\left(c\right)\right)}}-\underset{D_{c\hat{c}}}{\underset{︸}{H_{\theta_{c},\phi_{a}}\left(C|A\right)}}\;. \end{array}$$

### 3.3. Supervised and Semi-Supervised Models with/without Hand-Crafted Priors

Summarizing the above variational decomposition of (3) with the terms (4) and (6), we will proceed with four practical scenarios.

*Supervised training without latent space regularization (***baseline)**: is based on term $D_{c\hat{c}}$ in (6)

(7) $$L_{S-\mathrm{NoReg}}^{\mathrm{HCP}}\left(\theta_{c},\phi_{a}\right)=D_{c\hat{c}}.$$

*Semi-supervised training without latent space regularization* is based on terms $D_{c\hat{c}}$ and $D_{c}$ in (6):

(8) $$L_{\mathrm{SS}-\mathrm{NoReg}}^{\mathrm{HCP}}\left(\theta_{c},\phi_{a}\right)=D_{c\hat{c}}+D_{c}.$$

*Supervised training with latent space regularization* is based on term $D_{c\hat{c}}$ in (6) and either term $D_{a|x}$ or $D_{a}$ or jointly $D_{a|x}$ and $D_{a}$ in (4):

(9) $$L_{S-\mathrm{Reg}}^{\mathrm{HCP}}\left(\theta_{c},\phi_{a}\right)=E_{p_{D}\left(x\right)}\left[D_{a|x}\right]+D_{a}+\beta_{c}D_{c\hat{c}}.$$

*Semi-supervised training with latent space regularization* deploys all terms in (4) and (6):

(10) $$L_{\mathrm{SS}-\mathrm{Reg}}^{\mathrm{HCP}}\left(\theta_{c},\phi_{a}\right)=E_{p_{D}\left(x\right)}\left[D_{a|x}\right]+D_{a}+\beta_{c}D_{c\hat{c}}+\beta_{c}D_{c}.$$

The empirical evaluation of these setups on MNIST dataset is given in Section 5. The same architecture of encoder and decoder was used to establish the impact of each term in a function of available labeled data.

## 4. IB with Learnable Priors (LP)

In this section, we extend the results obtained for the hand-crafted priors to the learnable priors. Instead of applying the hand-crafted regularization of the latent representation a as suggested by the IB (3) and shown in Figure 1, we will assume that the latent representation a is regularized by an especially designed AE as shown in Figure 2. The AE-based regularization has two components: (i) the latent space z regularization and (ii) the observation space regularization. The design and training of this latent space regularizer in a form of the AE is guided by its own IB. In the general case, all elements of AE, i.e., its encoder-decoder pair, latent and observation space regularizers are conditioned by the learned class label c. The resulting Lagrangian with the learnable prior is (formally one should consider $I_{\phi_{a},\phi_{z},\theta_{c}}\left(X;Z|C\right)$ for the term A. However, since $I_{\phi_{a},\phi_{z},\theta_{c}}\left(X;Z|C\right)\leq I_{\phi_{a},\phi_{z},\theta_{c}}\left(A;Z|C\right)$ due to the Markovianity of considered architecture, we consider the decomposition starting from A [20], Data Processing Inequality, Theorem 2.8.1):

(11) $$L^{\mathrm{LP}}\left(\phi_{a},\phi_{z},\theta_{c},\theta_{x}\right)=\underset{A}{\underset{︸}{I_{\phi_{a},\phi_{z},\theta_{c}}\left(A;Z|C\right)}}-\beta_{x}\underset{B}{\underset{︸}{I_{\phi_{a},\phi_{z},\theta_{c},\theta_{x}}\left(X;Z|C\right)}}\;-\beta_{c}\underset{C}{\underset{︸}{I_{\phi_{a},\theta_{c}}^{L}\left(A;C\right)}},$$

where $\beta_{x}$ is a Lagrangian multiplier controlling the reconstruction of x at the decoder and $\beta_{c}$ is the same as in (1).

The terms A and B, conditioned by the class c, play a role of the latent space regularizer by imposing the learnable constrains on the vector a. These two terms correspond to the hand-crafted counterpart $I_{\phi_{a}}\left(X;A\right)$ in (3). The term C in the learnable IB formulation corresponds to the classification part of hand-crafted IB in (3) and can be factorized along the same lines as in (6). Therefore, we will proceed with the factorization of terms A and B.

One can also consider the following IB formulation with the learnable priors with no conditioning on c in term A in (11) leading to an unconditional counterpart D below that can be viewed as an IB generalization of semi-supervised AAE [2]:

(12) $$L_{\mathrm{AAE}}^{\mathrm{LP}}\left(\phi_{a},\phi_{z},\theta_{c},\theta_{x}\right)=\underset{D}{\underset{︸}{I_{\phi_{a},\phi_{z}}\left(A;Z\right)}}-\beta_{x}\underset{B}{\underset{︸}{I_{\phi_{a},\phi_{z},\theta_{c},\theta_{x}}\left(X;Z|C\right)}}\;-\beta_{c}\underset{C}{\underset{︸}{I_{\phi_{a},\theta_{c}}^{L}\left(A;C\right)}}.$$

### 4.1. Decomposition of Latent Space Regularizer

We will denote $p_{\phi_{a},\phi_{z},\theta_{c}}\left(x,a,c,z\right)=p_{D}\left(x\right)q_{\phi_{a}}\left(a|x\right)p_{\theta_{c}}\left(c|a\right)q_{\phi_{z}}\left(z|a,c\right)$ and decompose the term A in (11) using variational factorization as:

(13) $$\begin{array}{cc} I_{\phi_{a},\phi_{z},\theta_{c}}\left(A,Z|C\right) & =E_{p_{\phi_{a},\phi_{z},\theta_{c}}\left(x,a,c,z\right)}\left[\log\frac{q_{\phi_{z}}\left(z|a,c\right)}{q_{\phi_{z}}\left(z|c\right)}\frac{p_{\theta_{z}}\left(z\right)}{p_{\theta_{z}}\left(z\right)}\right]\\ & =E_{p_{D}\left(x\right)}\left[E_{q_{\phi_{a}}\left(a|x\right)}\left[E_{p_{\theta_{c}}\left(c|a\right)}\underset{D_{z|a,c}}{\underset{︸}{\left[D_{\mathrm{KL}}\left(q_{\phi_{z}}\left(z|A=a,C=c\right)∥p_{\theta_{z}}\left(z\right)\right)\right]}}\;\right]\right],\\ & -E_{p_{D}\left(x\right)}\left[E_{q_{\phi_{a}}\left(a|x\right)}\left[E_{p_{\theta_{c}}\left(c|a\right)}\underset{D_{z|c}}{\underset{︸}{\left[D_{\mathrm{KL}}\left(q_{\phi_{z}}\left(z|C=c\right)∥p_{\theta_{z}}\left(z\right)\right)\right]}}\;\right]\right], \end{array}$$

where $D_{z|a,c}≜D_{\mathrm{KL}}\left(q_{\phi_{z}}\left(z|a,c\right)∥p_{\theta_{z}}\left(z\right)\right)=E_{q_{\phi_{z}}\left(z|a,c\right)}\left[\log\frac{q_{\phi_{z}}\left(z|a,c\right)}{p_{\theta_{z}}\left(z\right)}\right]$ and $D_{z|c}≜D_{\mathrm{KL}}\left(q_{\phi_{z}}\left(z|c\right)∥p_{\theta_{z}}\left(z\right)\right)=E_{q_{\phi_{z}}\left(z|c\right)}\left[\log\frac{q_{\phi_{z}}\left(z|c\right)}{p_{\theta_{z}}\left(z\right)}\right]$ denote the KL-divergence terms and $q_{\phi_{z}}\left(z|c\right)=E_{p_{D}\left(x\right)}\left[E_{q_{\phi_{a}}\left(a|x\right)}\left[q_{\phi_{z}}\left(z|a,c\right))\right]\right]$.

### 4.2. Decomposition of Reconstruction Space Regularizer

Denoting $p_{\phi_{a},\phi_{z},\theta_{c},\theta_{x}}\left(x,a,c,z\right)=p_{D}\left(x\right)q_{\phi_{a}}\left(a|x\right)p_{\theta_{c}}\left(c|a\right)q_{\phi_{z}}\left(z|a,c\right)p_{\theta_{x}}\left(x|z,c\right)$, we decompose the term B in (11) as:

(14) $$\begin{array}{cc} I_{\phi_{a},\phi_{z},\theta_{c},\theta_{x}}\left(X;Z|C\right) & =E_{p_{\phi_{a},\phi_{z},\theta_{c},\theta_{c}}\left(x,a,c,z\right)}\left[\log\frac{p_{\theta_{x}}\left(x|z,c\right)}{p_{D}\left(x|c\right)}\frac{p_{\theta_{x}}\left(x\right)}{p_{\theta_{x}}\left(x\right)}\right]\\ & =E_{p_{\theta_{c}}\left(c\right)}\left[H(p_{D}\left(x|c\right);p_{\theta_{x}}\left(x\right))\right]\\ & -E_{p_{\theta_{c}}\left(c\right)}\underset{D_{x|c}}{\underset{︸}{\left[D_{\mathrm{KL}}\left(p_{D}\left(x|C=c\right)∥p_{\theta_{x}}\left(x\right)\right)\right]}}\;-\underset{D_{x\hat{x}}}{\underset{︸}{H_{\phi_{a},\phi_{z},\theta_{c},\theta_{x}}\left(X|Z,C\right)}}\;, \end{array}$$

where $p_{\theta_{c}}\left(c\right)=E_{p_{D}\left(x\right)}\left[E_{q_{\phi_{a}}\left(a|x\right)}\left[p_{\theta_{c}}\left(c|a\right)\right]\right]$. The terms are defined as $H\left(p_{D}\left(x|c\right);p_{\theta_{x}}\left(x\right)\right)=-E_{p_{D}\left(x|c\right)}\left[\log p_{\theta_{x}}\left(x\right)\right]$, $D_{x|c}≜D_{\mathrm{KL}}\left(p_{D}\left(x|C=c\right)∥p_{\theta_{x}}\left(x\right)\right)=E_{p_{D}\left(x|c\right)}\left[\log\frac{p_{D}\left(x|c\right)}{p_{\theta_{x}}\left(x\right)}\right]$ and $D_{x\hat{x}}≜H_{\phi_{a},\phi_{z},\theta_{c},\theta_{x}}\left(X|Z,C\right)=-E_{p_{D}\left(x\right)}\left[E_{q_{\phi_{a}}\left(a|x\right)}\left[E_{p_{\theta_{c}}\left(c|a\right)}\left[E_{q_{\phi_{z}}\left(z|a,c\right)}\left[\log p_{\theta_{x}}\left(x|z,c\right)\right]\right]\right]\right]$. Since $E_{p_{\theta_{c}}\left(c\right)}\left[H(p_{D}\left(x|c\right);p_{\theta_{x}}\left(x\right))\right]\geq 0$, we can lower bound $I_{\phi_{a},\phi_{z},\theta_{c},\theta_{x}}\left(X;Z|C\right)\geq I_{\phi_{a},\phi_{z},\theta_{c},\theta_{x}}^{L}\left(X;Z|C\right)≜-D_{x|c}-D_{x\hat{x}}$.

### 4.3. Semi-Supervised Models with Learnable Priors

Summarizing the above variational decomposition of (11) with the terms (13) and (14), we will consider semi-supervised training with latent space regularization as:

(15) $$\begin{array}{cc} L_{\mathrm{SS}-\mathrm{Reg}}^{\mathrm{LP}}\left(\theta_{c},\theta_{x},\phi_{a},\phi_{z}\right) & =E_{p_{D}\left(x\right)}\left[E_{q_{\phi_{a}}\left(a|x\right)}\left[E_{p_{\theta_{c}}\left(c|a\right)}\left[D_{z|a,c}\right]\right]\right]+E_{p_{D}\left(x\right)}\left[E_{q_{\phi_{a}}\left(a|x\right)}\left[E_{p_{\theta_{c}}\left(c|a\right)}\left[D_{z|c}\right]\right]\right]\\ & +\beta_{x}D_{x\hat{x}}+\beta_{x}E_{p_{\theta_{c}}\left(c\right)}\left[D_{x|c}\right]+\beta_{c}D_{c\hat{c}}+\beta_{c}D_{c}. \end{array}$$

To create a link to the semi-supervised AAE [2], we also consider (12), where all latent and reconstruction space regularizers are independent of c, i.e., do not contain conditioning on c.

*Semi-supervised training with latent space regularization and MSE reconstruction* based on (12):

(16) $$L_{\mathrm{SS}-\mathrm{AAE}}^{\mathrm{LP}}\left(\theta_{c},\theta_{x},\phi_{a},\phi_{z}\right)=D_{z}+\beta_{x}D_{x\hat{x}}+\beta_{c}D_{c\hat{c}}+\beta_{c}D_{c},$$

where $D_{z}≜D_{\mathrm{KL}}\left(q_{\phi_{z}}\left(z\right)∥p_{\theta_{z}}\left(z\right)\right)=E_{q_{\phi_{z}}\left(z\right)}\left[\log\frac{q_{\phi_{z}}\left(z\right)}{p_{\theta_{z}}\left(z\right)}\right]$.

*Semi-supervised training with latent space regularization and with MSE and adversarial reconstruction* based on (12) deploys all terms:

(17) $$L_{\mathrm{SS}-{\mathrm{AAE}}_{\mathrm{complete}}}^{\mathrm{LP}}\left(\theta_{c},\theta_{x},\phi_{a},\phi_{z}\right)=D_{z}+\beta_{x}D_{x\hat{x}}+\beta_{x}D_{x}+\beta_{c}D_{c\hat{c}}+\beta_{c}D_{c},$$

where $D_{x}≜D_{\mathrm{KL}}\left(p_{D}\left(x\right)∥p_{\theta_{x}}\left(x\right)\right)=E_{p_{D}\left(x\right)}\left[\log\frac{p_{D}\left(x\right)}{p_{\theta_{x}}\left(x\right)}\right]$.

### 4.4. Links to State-Of-The-Art Models

The considered HCP and LP models can be linked with several state-of-the-art unsupervised models such VAE [21,22], β-VAE [23], AAE [2] and BIB-AE [15] and semi-supervised models such as AAE [2], CatGAN [3], VAE (M1 + M2) [1] and SeGMA [18].

#### 4.4.1. Links to Unsupervised Models

The proposed LP model (11) generalizes unsupervised models without the categorical latent representation. In addition, the unsupervised models in a form of the auto-encoder are used as a latent space regularizer in the LP setup. For these reasons, we will briefly consider four models of interest, namely VAE, β-VAE, AAE, and BIB-AE.

Before we proceed with the analysis, we will define an unsupervised IB for these models. We will assume the fused encoders $q_{\phi_{a}}\left(a|x\right)$ and $q_{\phi_{z}}\left(z|a\right)$ without conditioning on c in the inference model according to Figure 2. We also assume no conditionally on c in the generative model.

The Lagrangian of unsupervised IB is defined according to [15]:

(18) $$\begin{array}{cc} L^{U_{L}}\left(\theta_{x},\phi_{z}\right) & =I_{\phi_{z}}\left(X;Z\right)-\beta_{x}I_{\phi_{z},\theta_{x}}\left(Z;X\right), \end{array}$$

where similarly to the supervised counterpart (4), we define the first term as:

(19) $$\begin{array}{cc} I_{\phi_{z}}\left(X;Z\right) & =E_{q_{\phi_{z}}\left(x,z\right)}\left[\log\frac{q_{\phi_{z}}\left(x,z\right)}{q_{\phi_{z}}\left(z\right)p_{D}\left(x\right)}\right]=E_{q_{\phi_{z}}\left(x,z\right)}\left[\log\frac{q_{\phi_{z}}\left(z|x\right)}{q_{\phi_{z}}\left(z\right)}\frac{p_{\theta_{z}}\left(z\right)}{p_{\theta_{z}}\left(z\right)}\right]\\ & =E_{p_{D}\left(x\right)}\underset{D_{z|x}}{\underset{︸}{\left[D_{\mathrm{KL}}\left(q_{\phi_{z}}\left(z|X=x\right)∥p_{\theta_{z}}\left(z\right)\right)\right]}}\;-\underset{D_{z}}{\underset{︸}{D_{\mathrm{KL}}\left(q_{\phi_{z}}\left(z\right)∥p_{\theta_{z}}\left(z\right)\right)}}\;, \end{array}$$

and similarly to (14) the second term is defined as:

(20) $$\begin{array}{cc} I_{\phi_{z},\theta_{x}}\left(Z;X\right) & =E_{p_{D}\left(x\right)}\left[E_{q_{\phi_{z}}\left(z|x\right)}\left[\log\frac{p_{\theta_{x}}\left(x|z\right)}{p_{D}\left(x\right)}\frac{p_{\theta_{x}}\left(x\right)}{p_{\theta_{x}}\left(x\right)}\right]\right]\\ & =H\left(p_{D}\left(x|c\right);p_{\theta_{x}}\left(x\right)\right)-\underset{D_{x}}{\underset{︸}{D_{\mathrm{KL}}\left(p_{D}\left(x\right)∥p_{\theta_{x}}\left(x\right)\right)}}\;-\underset{D_{x\hat{x}}}{\underset{︸}{H_{\phi_{z},\theta_{x}}\left(X|Z\right)}}\;, \end{array}$$

where the definition of all terms should follow from the above equations. Since $H(p_{D}\left(x|c\right);p_{\theta_{x}}\left(x\right))\geq 0$, we can lower bound $I_{\phi_{z},\theta_{x}}\left(Z;X\right)\geq -D_{x}-D_{x\hat{x}}$.

Having defined the unsupervised IB variational bounded decomposition, we can proceed with an analysis of the related state-of-the-art methods along the lines of analysis introduced in Summary part of Section 2.

**VAE** [21,22] and **β-VAE** [23]:

1. *The targeted tasks*: auto-encoding and generation.
2. *The architecture in terms of the latent space representation*: the encoder outputs two vectors representing the mean and standard deviation vectors that control a new latent representation $z=f_{\phi_{z}}\left(x\right)+\sigma_{\phi_{z}}\left(x\right)⊙\epsilon$, where $f_{\phi_{z}}\left(x\right)$ and $\sigma_{\phi_{z}}\left(x\right)$ are outputs of the encoder and ϵ is assumed to be a zero mean unit variance Gaussian vector.
3. *The usage of IB or other underlying frameworks*: both VAE and β-VAE use evidence lower bound (ELBO) and are not derived from the IB framework. However, it can be shown [15] that the Lagrangian (18) can be reformulated for VAE and β−VAE as:
  (21) $$L_{\beta -\mathrm{VAE}}\left(\theta_{x},\phi_{z}\right)=E_{p_{D}\left(x\right)}\left[D_{z|x}\right]+\beta_{x}D_{x\hat{x}},$$
  where $\beta_{x}=1$ for VAE. It can be noted that the VAE and β-VAE are based on an upper bound on the mutual information term $I_{\phi_{z}}\left(X;Z\right)\leq E_{p_{D}\left(x\right)}\left[D_{z|x}\right]$, since $D_{\mathrm{KL}}\left(q_{\phi_{z}}\left(z\right)∥p_{\theta_{z}}\left(z\right)\right)\geq 0$. Similar considerations apply to the second term since $D_{\mathrm{KL}}\left(p_{D}\left(x\right)∥p_{\theta_{x}}\left(x\right)\right)\geq 0$.
4. *The label space regularization*: does not apply here due to the unsupervised setting.
5. *The latent space regularization*: is based on the hand-crafted prior with Gaussian pdf.
6. *The reconstruction space regularization in case of reconstruction loss*: is based on the mean square error (MSE) counterpart of $D_{x\hat{x}}$ that corresponds to the Guassian likelihood assumption.

**Unsupervised AAE** [2]:

1. *The targeted tasks*: auto-encoding and generation.
2. *The architecture in terms of the latent space representation*: the encoder outputs one vector in stochastic or deterministic way as $z=f_{\phi_{z}}\left(x\right)$.
3. *The usage of IB or other underlying frameworks*: AAE is not derived from the IB framework. As shown in [15], the AAE equivalent Lagrangian (18) can be linked with the IB formulation and defined as:
  (22) $$L_{\mathrm{AAE}}\left(\theta_{x},\phi_{z}\right)=D_{z}+\beta_{x}D_{x\hat{x}},$$
  where $\beta_{x}=1$ in the original AAE formulation. It should be pointed out that the IB formulation of AAE contains the term $D_{x\hat{x}}$, whose origin can be explained in the same way as for the VAE. Despite the fact that the term $D_{z}$ indeed appears in (22) with the opposite sign, it cannot be interpreted either as an upper bound on $I_{\phi_{z}}\left(X;Z\right)$ similarly to the VAE or as a lower bound. The goal of AAE is to minimize the reconstruction loss or to maximize the log-likelihood by ensuring that the latent space marginal distribution $q_{\phi_{z}}\left(z\right)$ matches the prior $p_{\theta_{z}}\left(z\right)$. The latter corresponds to the minimization of $D_{\mathrm{KL}}\left(q_{\phi_{z}}\left(z\right)∥p_{\theta_{z}}\left(z\right)\right)$, i.e., $D_{z}$ term.
4. *The label space regularization*: does not apply here due to the unsupervised setting.
5. *The latent space regularization*: is based on the hand-crafted prior with zero mean unit variance Gaussian pdf for each dimension.
6. *The reconstruction space regularization in case of reconstruction loss*: is based on the MSE.

**BIB-AE** [15]:

1. *The targeted tasks*: auto-encoding and generation.
2. *The architecture in terms of the latent space representation*: the encoder outputs one vector using any form of stochastic or deterministic encoding.
3. *The usage of IB or other underlying frameworks*: the BIB-AE is derived from the unsupervised IB (18) and its Lagrangian is defined as:
  (23) $$L_{\mathrm{BIB}-\mathrm{AE}}\left(\theta_{x},\phi_{z}\right)=E_{p_{D}\left(x\right)}\left[D_{z|x}\right]-D_{z}+\beta_{x}D_{x}+\beta_{x}D_{x\hat{x}}.$$
4. *The label space regularization*: does not apply here due to the unsupervised setting.
5. *The latent space regularization*: is based on the hand-crafted prior with Gaussian pdf applied to both conditional and unconditional terms. In fact, the prior for $D_{z}$ can be any but $D_{z|x}$ requires analytical parametrisation.
6. *The reconstruction space regularization in case of reconstruction loss*: is based on the MSE counterpart of $D_{x\hat{x}}$ and the discriminator $D_{x}$. This is a disctintive feature in comparison to VAE and AAE.

In summary, BIB-AE includes VAE and AAE as two particular cases. In turns, it should be clear that the regularizer of semi-supervised model considered in this paper resembles the BIB-AE model and extends it to the conditional case that will be considered below.

#### 4.4.2. Links to Semi-Supervised Models

The proposed LP model (11) is also related to several state-of-the-art semi-supervised models used for the classification. As pointed out in the introduction, we only consider available labeled and unlabeled samples in our analysis. The extension to the augmented samples, i.e., permutations, syntehtically generated samples, i.e., fakes, and the adversarial examples for both latent space and label space regularizations can be performed along the line of analysis but it goes beyond the scope and focus of this paper.

**Semi-supervised AAE** [2]:

1. *The targeted tasks*: auto-encoding, clustering, (conditional) generation and classification.
2. *The architecture in terms of the latent space representation*: the encoder outputs two vectors representing the discrete class and continuous type of style. The class distribution is assumed to follow categorical distribution and style Gaussian one. Both constraints on the prior distributions are ensured using adversarial framework with two corresponding discriminators. In its original setting, AAE does not use any augmented samples or adversarial examples.
  *Remark*: It should be pointed out that in our architecture we consider the latent space to be represented by the vector a, which is fed to the classifier and regularizer that gives a natural consideration of IB and corresponding regularization and priors. In the case of semi-supervised AAE, the latent space is considered by the class and style representations directly. Therefore, to make it coherent with our case, one should assume that the class vector of semi-supervised AAE corresponds to the vector c and the style vector to the vector z.
3. *The usage of IB or other underlying frameworks*: AAE is not derived from the IB framework. However, as shown in our analysis the semi-supervised AAE represents the learnable prior case in part of latent space regularization. The corresponding Lagrangian of semi-supervised AAE is given by (16) and considered in Section 4.3.
4. *The label space regularization*: is based on the adversarial discriminator in assumption that the class labels follow categorical distribution. This is applied to both labeled and unlabeled samples.
5. *The latent space regularization*: is based on the learnable prior with Gaussian pdf of AE.
6. *The reconstruction space regularization in case of reconstruction loss*: is only based on the MSE.

**CatGAN** [3]: is based on an extension of classical GAN binary discriminator designed to distinguish between the original images and fake images generated from the latent space distribution to a multi-class discriminator. The author assumes the one-hot-vector encoding of class labels. The system is considered for the unsupervised and semi-supervised modes. For both modes the one-hot-vector encoding is used to encoded class labels. For the unsupervised mode, the system has an access only to the unlabeled data and the output of the classifier is considered to be a clustering to a predefined number of clusters/classes. The main idea behind the unsupervised training consists of a training of the discriminator that any sample from the set of original images is assigned to one of the classes with high fidelity whereas any fake or adversarial sample is assigned to all classes almost equiprobably. This corresponds to the fake samples and the regularization in the label space is based on the considered and extended framework of entropy minimization-based regularization. In the case of absence of fakes, this regularization coincides with the semi-supervised AAE label space regularization under the categorical distribution and adversarial discriminator that is equivalent to enforcing the minimum entropy of label space. However, the encoding of fake samples is equivalent to a sort of rejection option expressed via the activation of classes that have maximum entropy or uniform distribution over the classes. Equivalently, the above types of encoding can be considered to be the maximization of mutual information between the original data and encoded class labels and minimization of mutual information between the fakes/adversarial samples and the class labels. Semi-supevised CatGAN model adds a cross-entropy term computed for the true labeled samples.

Therefore, in summary:

1. *The targeted tasks*: auto-encoding, clustering, generation and classification.
2. *The architecture in terms of the latent space representation*: there is no encoder as such and instead the system has a generator/decoder that generates samples from a random latent space a following some hand-crafted prior. The second element of architecture is a classifier with the min/max entropy optimization for the original and fake samples. The encoding of classes is assumed to be a one-hot-vector encoding.
3. *The usage of IB or other underlying frameworks*: CatGAN is not derived from the IB framework. However, as shown in [15], one can apply the IB formulation to the adversarial generative models as in the case of CatGAN assuming that the term $I_{\phi_{a}}\left(X;A\right)=0$ in (3) due to the absence of encoder as such. The minimization problem (3) reduces to the maximization of the second term $I_{\phi_{a},\theta_{c}}\left(A;C\right)$ expressed via its lower bound of variational decomposition (6). The first term $D_{c}$ enforces that the class labels of unlabeled samples follow the defined prior distribution p(c) with the above property of entropy minimization under one-hot-vector encoding whereas the second term $D_{c\hat{c}}$ reflects the supervised part for labeled samples. In the original CatGAN formulation, the author does not use the expression for the mutual information for the decoder/generator training as it is shown above but instead uses the decomposition of mutual information via the difference of corresponding entropies (see, first two terms in (9) in [3]). As we have pointed out, we do not include in our analysis the term corresponding to the fake samples as in original CatGAN. However, we do believe that this form of regularization does play an important role for the semi-supervised classification. The impact of this terms requires additional studies.
4. *The label space regularization*: is based on the above assumptions for labeled samples, which are included into the cross-entropy term, unlabeled samples included into the entropy minimization term and fake samples included into the entropy maximization term in the original CatGAN method.
5. *The latent space regularization*: is based on the hand-crafted prior.
6. *The reconstruction space regularization in case of reconstruction loss*: is based on the adversarial discriminator only.

**SeGMA** [18]: is a semi-supervised clustering and generative system with a single latent vector representation auto-encoder similar in spirit to the unsupervised version of AAE that can be also used for the classification. The latent space of SeGMA is assumed to follow a mixture of Gaussians. Using a small labeled data set, classes are assigned to components of this mixture of Gaussians by minimizing the cross-entropy loss induced by the class posterior distribution of a simple Gaussian classifier. The resulting mixture describes the distribution of the whole data, and representatives of individual classes are generated by sampling from its components. In the classification setup, SeGMA uses the latent space clustering scheme for the classification.

Therefore, in summary:

1. *The targeted tasks*: auto-encoding, clustering, generation and classification.
2. *The architecture in terms of the latent space representation*: a single vector representation following mixture of Gaussians distribution.
3. *The usage of IB or other underlying frameworks*: SeGMA is not derived from the IB framework but a link to the regularized ELBO an other related auto-encoders with interpretable latent space is demonstrated. However, as in previous methods it can be linked to the considered IB interpretation of the semi-supervised methods with hand-crafted priors (16). An equivalent Lagrangian of SeGMA is:
  (24) $$L_{\mathrm{SeGMA}}\left(\theta_{c},\theta_{x},\phi_{z}\right)=D_{z}+\beta_{x}D_{x\hat{x}}+\beta_{c}D_{c\hat{c}},$$
  where the latent space discriminator $D_{z}$ is assumed to be the maximum mean discrepancy (MMD) penalty that is analytically defined for the mixture of Gaussians pdf, $D_{x\hat{x}}$ is represented by the MSE and $D_{c\hat{c}}$ represents the cross-entropy for the labeled data defined over class labels deduced from the latent space representation.
4. *The label space regularization*: is based on the above assumptions for labeled samples, which are included into the cross-entropy term as discussed above.
5. *The latent space regularization*: is based on the hand-crafted mixture of Gaussians pdf.
6. *The reconstruction space regularization in case of reconstruction loss*: is based on the MSE.

**VAE (M1 + M2)** [1]: is based on the combination of several models. The model M1 represents a vanilla VAE considered in Section 4.4.1. Therefore, model M1 is a particular case of considered unsupervised IB. The model M2 is a combination of encoder producing a continuous latent representation and following Gaussian distribution and a classifier that takes as an input original data in parallel to the model M1. The class labels are encoded using the one-hot-vector representations and follow categorical distribution with a hyper-parameter following the symmetric Dirichlet distribution. The decoder of model M2 takes as an input the continuous latent representation and output of classifier. The decoder is trained under the MSE distortion metric. It is important to point out that the classifier works with the input data directly but not with the common latent space such as in the considered LP model. For this reason, it is an obvious analogy with the considered LP model (11) under the assumption that a=x and all performed IB analysis directly applies to. However, as pointed by the authors, the performance of model M2 in the semi-supervised classification for the limited number of labeled samples is relatively poor. That is why the third hybrid model M1 + M2 is considered when the models M1 and M2 and used in a stacked way. At the first stage, the model M1 is learned as the usual VAE. Then the latent space of model M1 is used as an input to the model M2 trained in a semi-supervised way. Such a two-stage approach closely resembles the learnable prior architecture presented in Figure 2. However, our model is end-to-end trained with the explainable common latent space and IB origin, while the model M1 + M2 is trained in two stages with the use of regularized ELBO for the derivation of model M2.

1. *The targeted tasks*: auto-encoding, clustering, (conditional) generation and classification.
2. *The architecture in terms of the latent space representation*: the stacked combination of models M1 and M2 is used as discussed above.
3. *The usage of IB or other underlying frameworks*: VAE M1 + M2 is not derived from the IB framework but it is linked to the regularized ELBO with the cross-entropy for the labeled samples. The corresponding IB Lagrangian of semi-supervised VAE M1 + M2 under the assumption of end-to-end training can be defined as:
  (25) $$L_{\mathrm{SS}-\mathrm{VAE}\;M1+M2}^{\mathrm{LP}}\left(\theta_{c},\theta_{x},\phi_{a},\phi_{z}\right)=E_{p_{D}\left(x\right)}\left[D_{z|x}\right]+\beta_{x}D_{x\hat{x}}+\beta_{c}D_{c\hat{c}}+\beta_{c}D_{c}.$$
4. *The label space regularization*: is based on the assumption of categorical distribution of labels.
5. *The reconstruction space regularization in case of reconstruction loss*: is only based on the MSE.

## 5. Experimental Results

### 5.1. Experimental Setup

The tested system is based on (i) the deterministic encoder and decoder, (ii) the stochastic encoder of type $a=f_{\phi_{a}}\left(x+\epsilon \right)$ with the data independent perturbations ϵ and deterministic decoder. The density ratio estimator [24] is used to measure all KL-divergences. The results of semi-supervised classification on the MNIST dataset are reported in Table 1, where symbol *D* indicates the deterministic setup (i) and symbol *S* corresponds to the stochastic one (ii). To choose the optimal parameters of systems, e.g., the Lagrangian multipliers in the considered models, we used 3-run cross-validation with the randomly chosen labeled examples as shown in Appendix B, Appendix C, Appendix D, Appendix E, Appendix F and Appendix G. Once the model parameters were chosen, we run 10 time cross-validation and the average results are shown in Table 1.

Additionally, we performed a 10-run cross-validation on the SVHN dataset [25]. We used the same architecture as for MNIST with the same encoders, decoders and discriminators. In contrast to VAE M1 + M2, we used normalized raw data without any pre-processing. Additionally, in contrast to AAE, where an extra set of 531,131 unlabeled images was used for the semi-supervised training, in our experiments only a train set of 73,257 images was used for training. Moreover, the experiments were performed: (i) for the optimal parameters chosen after 3-run cross-validation for the MNIST dataset with no special adaption to SVHN dataset and (ii) under the network architectures with exactly the same number of used filters as given in Appendix B, Appendix C, Appendix D, Appendix E, Appendix F and Appendix G for the MNIST dataset. In summary, our goal is to test the generalization capacity of the proposed approach but not just to achieve the best performance by fine-tuning of network parameters. The obtained results are represented in Table 1.

We compare the considered architectures with several state-of-the-art semi-supervised methods such as AAE [2], CatGAN [3], VAE (M1 + M2) [1], IB multiview [5], MV-InfoMax [5] and InfoMax [3] with 100, 1000 and 60,000 training labeled samples. The expected training times for the considered models are given in Table 2. The source code is available at https://github.com/taranO/IB-semi-supervised-classification. The analysis of the latent space of trained models for the MNIST dataset is given in Appendix A.

### 5.2. Discussion MNIST

The deterministic and stochastic systems based on the learnable priors clearly demonstrate the state-of-the-art performance in comparison to the considered semi-supervised counterparts.

*Baseline Neural Network (NN):* the obtained results allow concluding that, if the amount of labeled training data is large, as shown in “all” column (Table 1), the latent space regularization has no practically significant impact on the classification performance for both hand crafted and learnable priors. The deep classifier is capable of learning a latent representation retaining only sufficient statistics in the latent space solely based on the cross-entropy component of IB classification term decomposition as shown in Table A1, row $D_{c\hat{c}}$ and column “all”. The classes appear to be well separable under this form of visualization. At the same time, the decrease of number of labeled samples leads to the degradation of classification accuracy as show in Table 1 for columns “1000” and “100”. This degradation is also clearly observed in Table A1, row $D_{c\hat{c}}$ and column “l00”, where there is larger overlap between the classes compared to the column “all”. The stochastic encoding via the addition of noise to the input samples does not enhance the performance with respect to the deterministic decoding for the small amount of labeled examples. One can assume that the presence of additive noise is not typical for the considered data, whereas the samples clearly differ in the geometrical appearance. Therefore, we can only assume that random geometrical permutations would be a more interesting alternative to the additive noise permutations/encoding.

*No priors on latent space:* to investigate the impact of unlabeled data, we add the adversarial regularizer $D_{c}$ to the baseline classifier based on $D_{c\hat{c}}$. The term $D_{c}$ enforces the distribution of class labels for the unlabeled samples to follow the categorical distribution. At this stage, no regularization of latent space is applied. The addition of the adversarial regularizer $D_{c}$, see “100” column (Table 1), allows reducing the classification error in comparison to the baseline classifier. Moreover, the stochastic encoder slightly outperforms the deterministic one for all numbers of labeled samples. However, the achieved classification error is far away from the performance of baseline classifier trained on the whole labeled data set. Thus, the cross-entropy and adversarial classification terms alone can hardly cope with the lack of labeled data, and proper regularization of the latent space is the main mechanism capable of retaining the most relevant representation.

*Hand crafted latent space priors:* along this line we investigate the impact of hand-crafted regularization in the form of the added discriminator $D_{a}$ imposing Gaussian prior on the latent representation a. The sole regularization of latent space with the hand-crafted prior on the Gaussianity does not reflect the complex nature of latent space of real data. As a result the performance of the regularized classifier $\beta_{c}D_{c\hat{c}}+D_{a}$ does not lead to a remarkable improvement in comparison to the non-regularized counterpart $D_{c\hat{c}}$ for both stochastic and deterministic types of encoding. When in addition the label space regularization $D_{c}$ is added to the final classifier $\beta_{c}D_{c\hat{c}}+D_{a}+\beta_{c}D_{c}$, it leads to the factor of 2 classification error reduction over the cross-entropy baseline classifier but it is still far away from the fully supervised baseline classifier trained on the fully labeled data set. At the same time, there is no significant difference between the stochastic and deterministic types of encoding.

*Learnable latent space priors:* along this line we will investigate the impact of learnable priors by adding the corresponding regularizations of the latent space of auto-encoder and data reconstruction. We investigate the role of reconstruction space regularization based on the MSE expressed via $D_{x\hat{x}}$ and joint $D_{x\hat{x}}$ and $D_{x}$. The addition of discriminator $D_{x}$ slightly enhances the classification but requires almost doubled training time as shown in Table 2. The stochastic encoding does not show any obvious advantage over the deterministic one in this setup. The separability of classes shown in Table A1, row $\beta_{c}D_{c\hat{c}}+\beta_{c}D_{c}+D_{z}+\beta_{x}D_{x\hat{x}}+\beta_{x}D_{x}$ and column ”l00”, is very close to those of column “all” and row $D_{c\hat{c}}$, i.e., the semi-supervised system with 100 labeled examples is capable of closely approximating the fully supervised one. We show the t-sne only for this setup since it practically coincides with $\beta_{c}D_{c\hat{c}}+\beta_{c}D_{c}+D_{z}+\beta_{x}D_{x\hat{x}}$. However, it should be pointed out that the learnable priors ensures the reconstruction of data from the compressed latent space and the learned representation is the sufficient statistics for the data reconstruction task but not for the classification one. Since the entropy of the classification task is significantly lower to those of reconstruction, such a learned representation contains more information than actually needed for the classification task. A fraction of retained information is irrelevant to the classification problem and might be a potential source of classification errors. This likely explains a gap in performance between the considered semi-supervised system and fully supervised one.

### 5.3. Discussion SVHN

In the SVHN test, we did not try to optimize the Lagrangian coefficients as it was done for MNIST. However, to compensate for a potential non-optimality, we perform the model training with the reduced learning rate as indicated in Table 2. As a result, the training time on the SVHN dataset is longer. Therefore, 10-run validation of the proposed framework on the SVHN dataset was done with the optimal Lagrangian multipliers determined on the MNIST dataset. In this respect, one might observe a small degradation of the obtained results compared to the state-of-the-art. Additionally, we did not apply any pre-processing such as PCA that was used in VAE M1 + M2 and we did not use the extended unlabeled dataset as it was done in case of AAE. One can clearly observe the same behavior of semi-supervised classifiers as for MNIST data set discussed in Section 5.2. Therefore, we can clearly confirm the role of learnable priors in the overall performance observed for both datasets.

## 6. Conclusions and Future Work

We have introduced a novel formulation of variational information bottleneck for semi-supervised classification. To overcome the problem of original bottleneck and to compensate the lack of labeled data in the semi-supervised setting, we considered two models of latent space regularization via hand-crafted and learnable priors. On a toy example of MNIST dataset we investigated how the parameters of proposed framework influence the performance of classifier. By end-to-end training, we demonstrate how the proposed framework compares to the state-of-the-art methods and approaches the performance of fully supervised classifier.

The envisioned future work is along the lines of providing a stronger compression yet preserving only classification task relevant information since retaining more task irrelevant information does not provide distinguishable classification features, i.e., it only ensures reliable data reconstruction. In this work, we have considered IB for the predictive latent space model. We think that the contrastive multi-view IB formulation would be an interesting candidate for the regularization of latent space. Additionally, we did not use the adversarially generated examples to impose the constraint on the minimization of mutual information between them and class labels or equivalently to maximize the entropy of class label distribution for these adversarial examples according to the framework of entropy minimization. This line of “adversarial” regularization seems to be a very interesting complement to the considered variational bottleneck. In this work, we considered a particular form of stochastic encoding by the addition of data independent noise to the input with the preservation of the same class labels. This also corresponds to the consistency regularization when samples can be more generally permuted including the geometrical transformations. It is also interesting to point out that the same form of generic permutations is used in the unsupervised constrastive loss-based multi-view formulations for the continual latent space representation as opposed to the categorical one in the consistency regularization. Finally, the conditional generation can be an interesting line of research considering the generation from discrete labels and continuous latent space of the autoencoder.

## Figures and Tables

**Table 1:** Semi-supervised classification performance (percentage error) for the optimal parameters (Appendix B, Appendix C, Appendix D, Appendix E, Appendix F and Appendix G) defined on the MNIST (*D*—deterministic; *S*—stochastic).

		MNIST (100)	MIST (1000)	MNIST (all)	SVHN (1000)
NN Baseline ($D_{c\hat{c}}$)	[*D*]	26.31 (±0.91)	7.50 (±0.19)	0.68 (±0.05)	36.16 (±0.77)
	[*S*]	26.78 (±1.66)	7.54 (±0.25)	0.70 (±0.05)	36.28 (±0.93)
InfoMax [3]	[*S*]	33.41	21.5	15.86	-
VAE [5]	[*S*]	14.26	8.71	5.02	-
MV-InfoMax [5]	[*S*]	13.22	7.39	6.07	-
IB multiview [5]	[*S*]	3.03	2.34	2.22	-
VAE (M1 + M2) [5]	[*S*]	3.33 (±0.14)	2.40 (±0.02)	0.96	36.02 (±0.10)
CatGAN	[*S*]	1.91 (±0.10)	1.73 (±0.18)	0.91	-
AAE	[*D*]	1.90 (±0.10)	1.60 (±0.08)	0.85 (±0.02)	17.70 (±0.30)
No priors on latent space					
$D_{c\hat{c}}+D_{c}$	[*D*]	20.72 (±1.58)	4.99 (±0.28)	0.69 (±0.04)	25.78 (±0.90)
	[*S*]	19.60 (±1.37)	4.49 (±0.25)	0.67 (±0.05)	26.34 (±0.80)
Hand crafted latent space priors					
$\beta_{c}D_{c\hat{c}}+D_{a}$	[*D*]	27.44 (±1.40)	6.77 (±0.34)	0.91 (±0.05)	35.94 (±1.08)
	[*S*]	27.48 (±1.07)	6.91 (±0.45)	0.88 (±0.05)	35.80 (±1.21)
$\beta_{c}D_{c\hat{c}}+D_{a}+\beta_{c}D_{c}$	[*D*]	12.04 (±4.46)	2.43 (±0.12)	0.81 (±0.05)	24.70 (±0.46)
	[*S*]	11.80 (±3.82)	2.40 (±0.10)	0.82 (±0.04)	24.62 (±0.54)
Learnable latent space priors					
$\beta_{c}D_{c\hat{c}}+\beta_{c}D_{c}+D_{z}+\beta_{x}D_{x\hat{x}}$	[*D*]	1.55 (±0.21)	1.25 (±0.10)	0.74 (±0.04)	20.07 (±0.36)
	[*S*]	1.49 (±0.18)	1.43 (±0.06)	0.78 (±0.04)	20.00 (±0.31)
$\beta_{c}D_{c\hat{c}}+\beta_{c}D_{c}+D_{z}+\beta_{x}D_{x\hat{x}}+\beta_{x}D_{x}$	[*D*]	1.38 (±0.09)	1.21 (±0.10)	0.77 (±0.06)	19.75 (±0.52)
	[*S*]	1.42 (±0.10)	1.16 (±0.09)	0.79 (±0.02)	19.71 (±0.26)

**Table 2:** Execution time (hours) per 100 epochs on one NVIDIA GPU. For the SVHN the models with the learnable latent space priors were trained with a learning rate 0.0001 that explains the longer time but without optimization of Lagrangians, i.e., the Lagrangians were re-used from pre-trained MNIST model. All the others models were trained with a learning rate 0.001.

	MNIST	SVHN
NN Baseline ($D_{c\hat{c}}$)	0.47–0.65	0.85–0.92
*No priors on latent space*	
$D_{c\hat{c}}+D_{c}$	0.47–0.65	0.85–0.92
*Hand crafted latent space priors*	
$\beta_{c}D_{c\hat{c}}+D_{a}$	0.47–0.65	1–1.05
$\beta_{c}D_{c\hat{c}}+D_{a}+\beta_{c}D_{c}$	0.97–1.18	1.5–1.6
*Learnable latent space priors*	
$\beta_{c}D_{c\hat{c}}+\beta_{c}D_{c}+D_{z}+\beta_{x}D_{x\hat{x}}$	1.23–1.6	2.25–2.3
$\beta_{c}D_{c\hat{c}}+\beta_{c}D_{c}+D_{z}+\beta_{x}D_{x\hat{x}}+\beta_{x}D_{x}$	1.98–2.42	3.5–3.55

**Figure A1:**
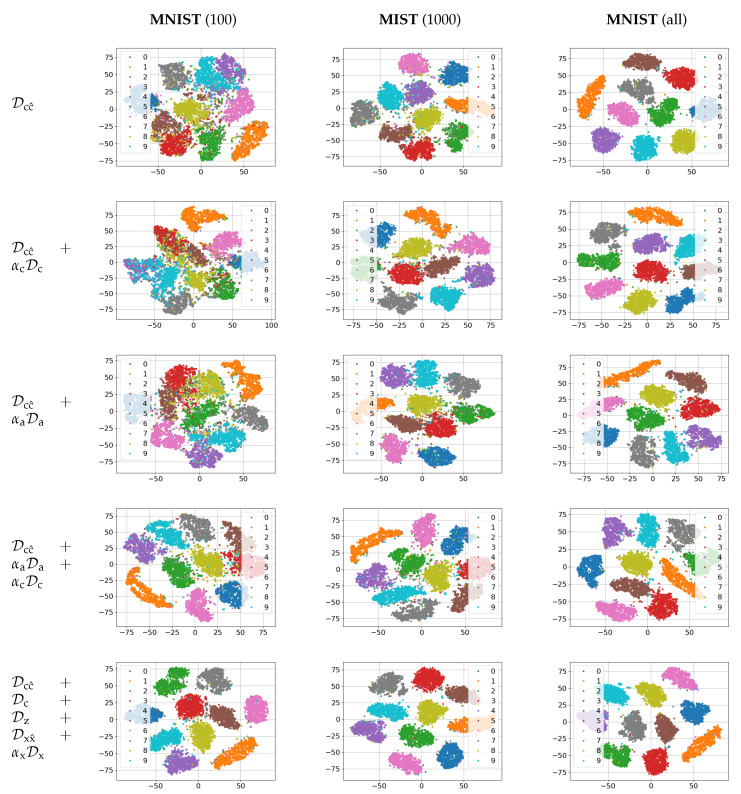
Latent space a (of size 1024) of classifier.

**Figure A2:**
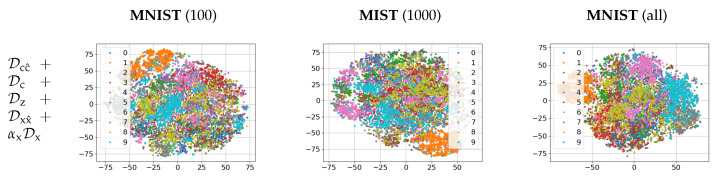
Latent space z (of size 20) of auto-encoder.

**Figure A3:**
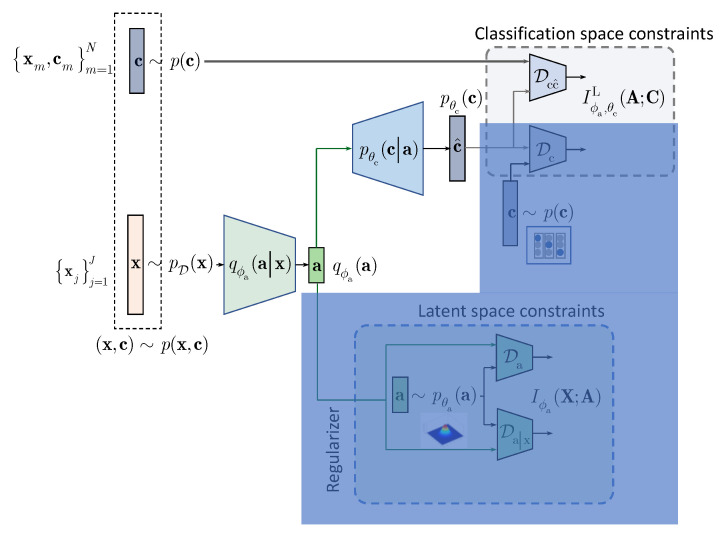
Baseline classifier based on $D_{c\hat{c}}$. The blue shadowed regions are not used.

**Figure A4:**
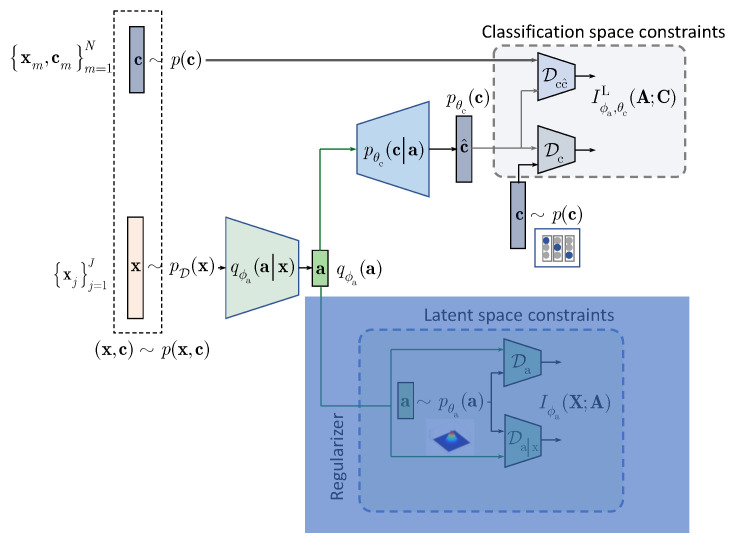
Semi-supervised classifier based on the cross-entropy $D_{c\hat{c}}$ and categorical class discriminator $D_{c}$. No latent space regularization is applied. The blue shadowed regions are not used.

**Figure A5:**
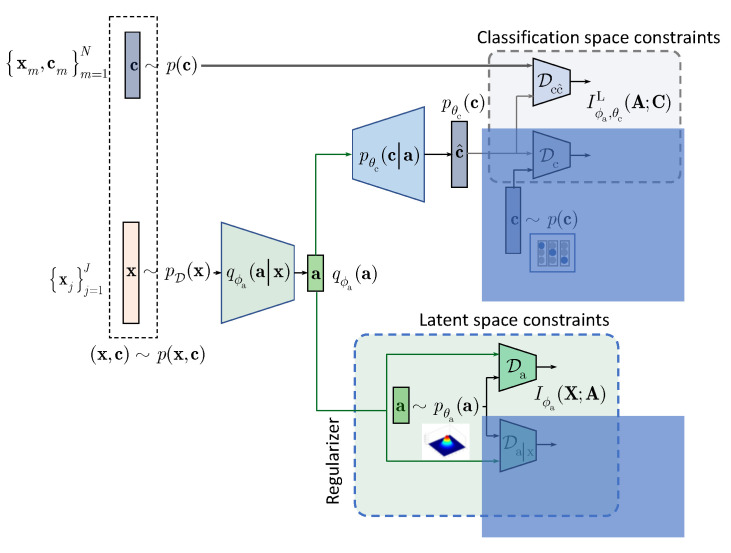
Supervised classifier based on the cross-entropy $D_{c\hat{c}}$ and hand crafted latent space regularization $D_{a}$. The blue shadowed parts are not used.

**Figure A6:**
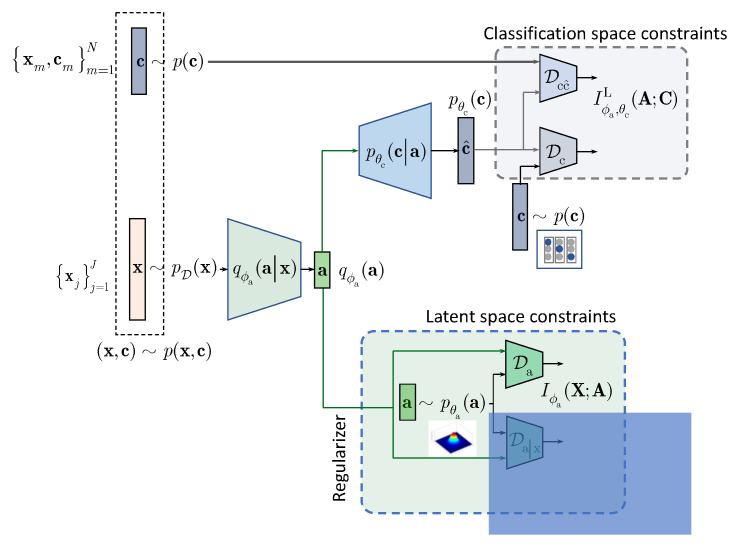
Semi-supervised classifier based on the cross-entropy $D_{c\hat{c}}$ and hand crafted latent space regularization $D_{a}$. The blue shadowed parts are not used.

**Figure A7:**
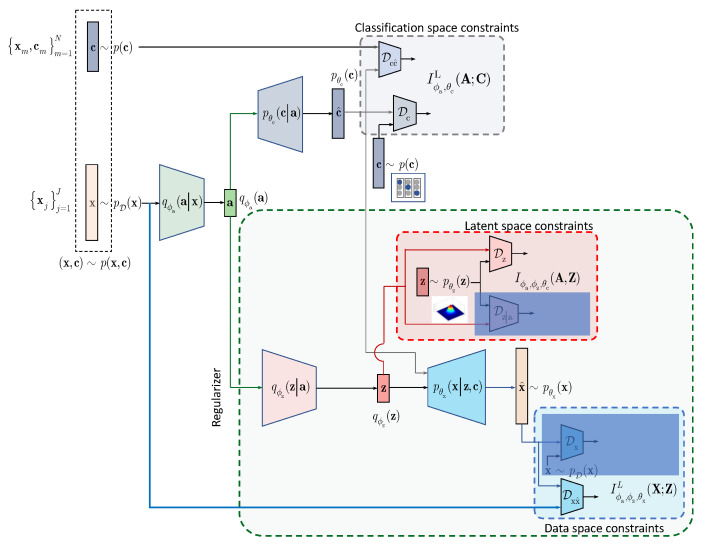
Semi-supervised classifier with learnable priors: the cross-entropy $D_{c\hat{c}}$, MSE $D_{x\hat{x}}$, class label $D_{c}$ and latent space regularization $D_{a}$. The blue shadowed parts are not used.

**Figure A8:**
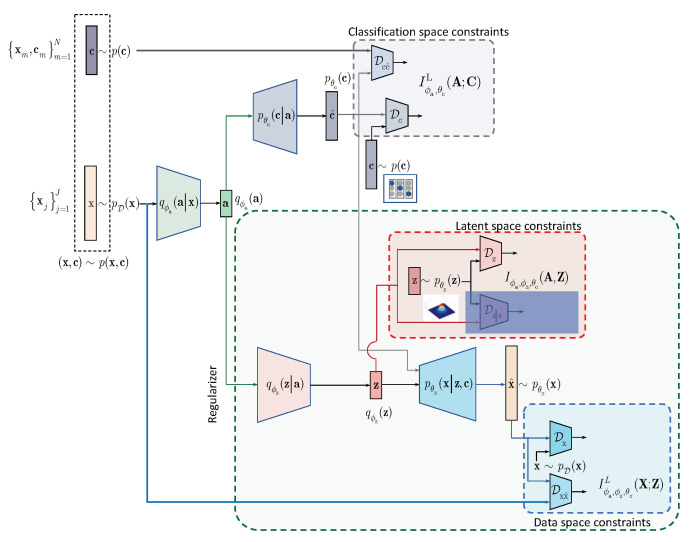
Semi-supervised classifier with learnable priors: the cross-entropy $D_{c\hat{c}}$, MSE $D_{x\hat{x}}$, adversarial reconstruction $D_{x}$, class label $D_{c}$ and latent space regularizer $D_{z}$. The blue shadowed parts are not used.

## Abbreviations

The following abbreviations are used in this manuscript:

IB	Information bottleneck
VAE	Variational autoencode
AAE	Adversarial autoencoder
CatGAN	Categorical generative adversarial networks
KL-divergences	Kullback–Leibler divergences
MSE	Mean squared error
HCP	IB with hand-crafted priors
LP	IB with learnable priors
NN	Neural Network
SS	Semi-supervised

## Appendix A. Latent Space of Trained Models

In this section, we consider the properties of classifier’s latent space for both the hand-crafted and learnable priors under different amount of training samples. Figure A1 and Figure A2 show t-sne plots for the perplexity 30 for 100, 1000 and 60,000 (“all”) training labels of the MNIST dataset.

The first raw of Figure A1 with the label “$D_{c\hat{c}}$” corresponds to the classifier considered in Appendix B. The latent space a of the classifier with “all” labels demonstrates the perfect separability of classes. The classes are far away from each other and there are practically no outliers leading to the misclassification. The decrease of the number of labels in the supervised setup, see the columns 1000 and 100, leads to a visible degradation of separability between the classes.

The regularization of class label space by the regularizer $D_{c}$ or by the hand-crafted latent space regularizer $D_{a}$ shown in raws “$D_{c\hat{c}}+\alpha_{c}D_{c}$” considered in Appendix C and “$D_{c\hat{c}}+\alpha_{a}D_{a}$” considered in Appendix D for the small number of training samples equal 100 does not significantly enhance the class separability with respect to “$D_{c\hat{c}}$”.

At the same time, the joint usage of the above regularizers according to the model “$D_{c\hat{c}}+\alpha_{c}D_{c}+\alpha_{a}D_{a}$” according to the model in Appendix E leads to the better separability of classes for 100 labels in comparison with the previous cases. At the same time, the addition of these regularizers does not have any impact on the latent space for “all” label case.

The introduction of learnable regularization of latent space along with the class label regularization according to the model “$D_{c\hat{c}}+D_{c}+D_{z}+D_{x\hat{x}}+\alpha_{x}D_{x}$” considered in Appendix G enhances the class separability in the latent space of classifier for 100 label case that is also very close to the fully supervised case.

For the comparison reasons, we also visualize the latent space of the auto-encoder z for the above model in Figure A2.

## Appendix B. Supervised Training without Latent Space Regularization (Baseline)

The baseline architecture is based on the cross-entropy term $D_{c\hat{c}}$ (7) in the main part of paper and depicted in Figure A3:

(A1) $$L_{S-\mathrm{NoReg}}^{\mathrm{HCP}}\left(\theta_{c},\phi_{a}\right)=D_{c\hat{c}}.$$

The parameters of encoder and decoder are shown in Table A1. The performance of baseline supervised classifier with and without batch normalization corresponds to the parameter $\alpha_{c}=0$ in Table A3 (deterministic scenario) and Table A4 (stochastic scenario).

**Table A1:** The network parameters of baseline classifier trained on $D_{c\hat{c}}$. The encoder is trained with and without batch normalization (BN) after Conv2D layers.

Encoder	
Size	Layer
28 × 28 × 1	Input
14 × 14 × 32	Conv2D, LeakyReLU
7 × 7 × 64	Conv2D, LeakyReLU
4 × 4 × 128	Conv2D, LeakyReLU
2048	Flatten
1024	FC, ReLU
**Decoder**	
Size	Layer
1024	Input
500	FC, ReLU
10	FC, Softmax

## Appendix C. Semi-Supervised Training without Latent Space Regularization and with Class Label Regularizer

This model is based on terms $D_{c\hat{c}}$ and $D_{c}$ in (8) in the main part of paper and schematically shown in Figure A4:

(A2) $$L_{\mathrm{SS}-\mathrm{NoReg}}^{\mathrm{HCP}}\left(\theta_{c},\phi_{a}\right)=D_{c\hat{c}}+\alpha_{c}D_{c}.$$

The parameters of encoder, decoder and discriminator are shown in Table A2. The KL-divergence term $D_{c}$ is implemented in a form of density ratio estimator (DRE). In the considered practical implementation, the parameter $\alpha_{c}$ controls the trade-off between the cross-entropy and class discriminator terms. The discriminator $D_{c}$ is trained in an adversarial way based on samples generated by the decoder and from targeted distribution.

The performance of semi-supervised classifier with and without batch normalization is shown in Table A3 (deterministic scenario) and Table A4 (stochastic scenario).

**Table A2:** The network parameters of semi-supervised classifier trained on $D_{c\hat{c}}$ and $D_{c}$. The encoder is trained with and without batch normalization (BN) after Conv2D layers.

Encoder	
Size	Layer
28 × 28 × 1	Input
14 × 14 × 32	Conv2D, LeakyReLU
7 × 7 × 64	Conv2D, LeakyReLU
4 × 4 × 128	Conv2D, LeakyReLU
2048	Flatten
1024	FC, ReLU
**Decoder**	
Size	Layer
1024	Input
500	FC, ReLU
10	FC, Softmax
$D_{c}$	
Size	Layer
10	Input
500	FC, ReLU
500	FC, ReLU
1	FC, Sigmoid

**Table A3:** The performance (percentage error) of **deterministic** classifier based on $D_{c\hat{c}}+\alpha_{c}D_{c}$ for the encoder with and without batch normalization as a function of Lagrangian multiplier $\alpha_{c}$ and the number of labelled examples.

Encoder Model	$\alpha_{c}$	Runs			Mean	std
		1	2	3		
**MNIST** 100						
without BN	0	26.56	26.24	28.04	26.95	0.96
	0.005	20.44	21.93	18.98	20.45	1.48
	0.0005	18.55	20.43	20.59	**19.86**	1.14
	1	19.23	22.42	20.57	20.74	1.60
with BN	0	29.37	29.27	30.62	29.75	0.75
	0.005	27.97	28.02	26.27	27.42	1.00
	0.0005	25.99	23.70	24.47	**24.72**	1.17
	1	27.78	31.98	35.88	31.88	4.05
**MNIST** 1000						
without BN	0	7.74	6.99	6.97	7.23	0.44
	0.005	5.62	6.06	5.60	**5.76**	0.26
	0.0005	6.30	6.12	6.02	6.15	0.14
	1	5.99	6.27	6.28	6.18	0.16
with BN	0	7.45	6.95	7.52	7.31	0.31
	0.005	5.57	5.08	5.22	**5.29**	0.25
	0.0005	5.60	6.05	6.22	5.96	0.32
	1	6.05	6.41	5.82	6.09	0.30
**MNIST** all						
without BN	0	0.83	0.83	0.74	**0.80**	0.05
	0.005	0.83	0.82	0.88	0.84	0.03
	0.0005	0.86	0.92	0.82	0.87	0.05
	1	0.72	0.85	0.87	0.81	0.08
with BN	0	0.73	0.67	0.79	0.73	0.06
	0.005	0.72	0.73	0.70	0.72	0.02
	0.0005	0.75	0.77	0.72	0.75	0.03
	1	0.67	0.68	0.73	**0.69**	0.03

**Table A4:** The performance (percentage error) of **stochastic** classifier with supervised noisy data (noise std = 0.1, # noise realisation = 3) based on $D_{c\hat{c}}+\alpha_{c}D_{c}$ for the encoder with and without batch normalization as a function of Lagrangian multiplier $\alpha_{c}$ and the number of labelled examples.

Encoder Model	$\alpha_{c}$	Runs			Mean	std
		1	2	3		
**MNIST** 100						
without BN	0	25.75	26.61	26.59	26.32	0.49
	0.005	23.34	21.38	24.37	23.03	1.52
	0.0005	19.92	15.83	16.03	**17.26**	2.31
	1	22.51	20.48	21.28	21.42	1.02
with BN	0	30.26	31.24	29.3	30.27	0.97
	0.005	21.17	24.41	24.75	23.44	1.98
	0.0005	22.97	26.38	24.44	24.60	1.71
	1	26.62	30.43	28.44	28.50	1.91
**MNIST** 1000						
without BN	0	7.68	7.30	7.23	7.4	0.24
	0.005	5.59	5.16	5.80	5.52	0.33
	0.0005	5.59	6	5.84	5.81	0.21
	1	6.66	6.8	7.62	7.03	0.52
with BN	0	6.97	7.06	7.66	7.23	0.38
	0.005	4.42	4.54	4.08	**4.35**	0.24
	0.0005	5.28	5.56	5.14	5.33	0.21
	1	5.77	5.88	5.72	5.79	0.08
**MNIST** all						
without BN	0	0.8	0.91	0.87	0.86	0.06
	0.005	0.77	0.82	0.88	0.82	0.06
	0.0005	0.86	0.81	0.87	0.85	0.03
	1	0.93	0.85	0.92	0.90	0.04
with BN	0	0.65	0.67	0.71	0.68	0.03
	0.005	0.69	0.77	0.68	0.71	0.05
	0.0005	0.78	0.71	0.74	0.74	0.04
	1	0.71	0.64	0.62	**0.66**	0.05

## Appendix D. Supervised Training with Hand Crafted Latent Space Regularization

This model is based on the cross-entropy term $D_{c\hat{c}}$ and either term $D_{a|x}$ or $D_{a}$ or jointly $D_{a|x}$ and $D_{a}$ as defined by (9) in the main part of paper. In our implementation, we consider the regularization based on the adversarial term $D_{a}$ similar to AAE due to the flexibility of imposing different priors on the latent space distribution. The implemented system is shown in Figure A5 and the training is based on:

(A3) $$L_{S-\mathrm{Reg}}^{\mathrm{HCP}}\left(\theta_{c},\phi_{a}\right)=D_{c\hat{c}}+\alpha_{a}D_{a},$$

where $\alpha_{a}$ is a regularization parameter controlling a trade-off between the cross-entropy term and latent space regularization term. We have replaced the Lagrangians above with respect to (9) in the main part of paper and used it in front of $D_{a}$ in contrast to the original formulation (9). It is done to keep the term $D_{c\hat{c}}$ without a multiplier as the reference to the baseline classifier.

The parameters of encoder, decoder and discriminator are summarized in Table A5. The performance of this classifier without and with batch normalization is shown in Table A6 (deterministic scenario) and Table A7 (stochastic scenario).

**Table A5:** The network parameters of supervised classifier trained on $D_{c\hat{c}}$ and $D_{a}$. The encoder is trained with and without batch normalization (BN) after Conv2D layers. $D_{a}$ is trained in the adversarial way.

Encoder	
Size	Layer
28 × 28 × 1	Input
14 × 14 × 32	Conv2D, LeakyReLU
7 × 7 × 64	Conv2D, LeakyReLU
4 × 4 × 128	Conv2D, LeakyReLU
2048	Flatten
1024	FC
**Decoder**	
Size	Layer
1024	Input
500	FC, ReLU
10	FC, Softmax
$D_{a}$	
Size	Layer
1024	Input
500	FC, ReLU
500	FC, ReLU
1	FC, Sigmoid

**Table A6:** The performance (percentage error) of **deterministic** classifier based on $D_{c\hat{c}}+\alpha_{a}D_{a}$ for the encoder with and without batch normalization as a function of Lagrangian multiplier.

Encoder Model	$\alpha_{a}$	Runs			Mean	std
		1	2	3		
**MNIST** 100						
without BN	0	26.79	27.26	27.39	27.15	0.32
	0.005	28.05	25.95	30.72	28.24	2.39
	0.0005	26.67	27.69	28.46	**27.61**	0.89
	1	33.42	33.05	34.81	33.76	0.92
with BN	0	30.37	29.32	29.82	29.83	0.52
	0.005	28.02	31.49	30.80	**30.11**	1.84
	0.0005	34.54	31.92	29.82	31.09	2.36
	1	34.43	44.35	44.25	41.01	5.70
**MNIST** 1000						
without BN	0	7.16	8.12	7.55	7.61	0.48
	0.005	7.02	6.34	6.59	6.65	0.34
	0.0005	6.73	6.34	6.82	**6.63**	0.26
	1	9.49	9.93	10.56	9.99	0.54
with BN	0	7.39	7.83	7.92	**7.72**	0.28
	0.005	7.94	7.15	8.53	7.88	0.69
	0.0005	8.00	9.62	9.51	9.05	0.91
	1	15.79	14.88	13.71	14.79	1.04
**MNIST** all						
without BN	0	0.76	0.70	0.81	**0.76**	0.06
	0.005	1.07	1.03	1.13	1.08	0.05
	0.0005	0.84	0.78	0.89	0.84	0.06
	1	4.78	7.24	4.71	5.58	1.44
with BN	0	0.68	0.68	0.69	**0.68**	0.01
	0.005	0.90	0.81	1.12	0.94	0.16
	0.0005	0.87	0.80	0.89	0.85	0.05
	1	2.37	3.61	4.35	3.44	1.00

**Table A7:** The performance (percentage error) of **stochastic** classifier with supervised noisy data (noise std = 0.1, # noise realisation = 3) based on $D_{c\hat{c}}+\alpha_{a}D_{a}$ for the encoder with and without batch normalization as a function of Lagrangian multiplier.

Encoder Model	$\alpha_{a}$	Runs			Mean	std
		1	2	3		
**MNIST** 100						
without BN	0.005	28.13	25.16	29.9	**27.73**	2.40
	0.0005	28.05	30.03	28.11	28.73	1.13
	1	32.33	34.09	33.73	33.38	0.93
with BN	0.005	32.25	33.47	26.01	30.58	4.00
	0.0005	33.37	36.15	35.65	35.06	1.48
	1	33.37	42.37	32.46	36.07	5.48
**MNIST** 1000						
without BN	0.005	7.37	7.17	6.65	7.06	0.37
	0.0005	7.48	6.68	6.67	**6.94**	0.46
	1	9.48	9.94	11.61	10.34	1.12
with BN	0.005	7.82	7.97	7.81	7.87	0.09
	0.0005	9.5	8.68	9.37	9.18	0.44
	1	12.99	10.52	9.98	11.16	1.60
**MNIST** all						
without BN	0.005	1.19	1.09	1.06	1.11	0.07
	0.0005	0.79	0.88	0.82	0.83	0.05
	1	6.22	4.81	5	5.34	0.77
with BN	0.005	0.94	1.07	1.04	1.02	0.07
	0.0005	0.78	0.81	0.78	**0.79**	0.02
	1	4.49	3.35	2.18	3.34	1.16

## Appendix E. Semi-Supervised Training with Hand Crafted Latent Space and Class Label Regularizations

This model is based on the cross-entropy term $D_{c\hat{c}}$ and either term $D_{a|x}$ or $D_{a}$ or jointly $D_{a|x}$ and $D_{a}$ and the label class regularizer $D_{c}$ as defined by (10) in the main part of paper. In our implementation, we consider the regularization based on the adversarial term $D_{a}$ only as shown in Figure A6. The training is based on:

(A4) $$L_{S-\mathrm{Reg}}^{\mathrm{HCP}}\left(\theta_{c},\phi_{a}\right)=D_{c\hat{c}}+\alpha_{c}D_{c}+\alpha_{a}D_{a}.$$

The parameters of encoder, decoder and both discriminators are shown in Table A8. The performance of this classifier without and with batch normalization is shown in Table A9 (deterministic scenario) and Table A10 (stochastic scenario).

**Table A8:** The network parameters of semi-supervised classifier trained on $D_{c\hat{c}}$, $D_{a}$ and $D_{c}$. The encoder is trained with and without batch normalization (BN) after Conv2D layers. $D_{a}$ and $D_{c}$ are trained in the adversarial way.

Encoder	
Size	Layer
28 × 28 × 1	Input
14 × 14 × 32	Conv2D, LeakyReLU
7 × 7 × 64	Conv2D, LeakyReLU
4 × 4 × 128	Conv2D, LeakyReLU
2048	Flatten
1024	FC
**Decoder**	
Size	Layer
1024	Input
500	FC, ReLU
10	FC, Softmax
$D_{c}$	
Size	Layer
10	Input
500	FC, ReLU
500	FC, ReLU
1	FC, Sigmoid
$D_{a}$	
Size	Layer
1024	Input
500	FC, ReLU
500	FC, ReLU
1	FC, Sigmoid

**Table A9:** The performance (percentage error) of **deterministic** classifier based on $D_{c\hat{c}}+\alpha_{a}D_{a}+\alpha_{c}D_{c}$ for the encoder with and without batch normalization.

Encoder Model	$\alpha_{a}$	$\alpha_{c}$	Runs			Mean	std
			1	2	3		
**MNIST** 100							
without BN	0.005	0.005	21.39	18.12	18.34	19.28	1.83
	0.0005	0.0005	15.33	22.36	13.80	17.16	4.56
	0.005	0.0005	25.66	26.25	28.81	26.91	1.67
	0.0005	0.005	9.82	13.44	13.06	**12.11**	1.99
with BN	0.005	0.005	23.45	21.19	28.87	24.50	3.94
	0.0005	0.0005	28.57	19.06	26.37	24.67	4.98
	0.005	0.0005	26.18	26.18	25.49	25.95	0.40
	0.0005	0.005	8.96	13.82	14.76	12.52	3.11
**MNIST** 1000							
without BN	0.005	0.005	3.91	4.21	3.70	3.94	0.26
	0.0005	0.0005	3.54	3.72	3.54	3.60	0.10
	0.005	0.0005	6.19	5.80	7.31	6.43	0.78
	0.0005	0.005	2.80	2.82	2.83	2.82	0.02
with BN	0.005	0.005	3.30	2.94	2.93	3.06	0.21
	0.0005	0.0005	2.80	2.53	2.50	2.61	0.17
	0.005	0.0005	3.51	3.75	4.12	3.79	0.31
	0.0005	0.005	2.58	2.27	2.24	**2.37**	0.19
**MNIST** all							
without BN	0.005	0.005	1.04	1.07	1.07	1.06	0.02
	0.0005	0.0005	0.86	0.90	0.88	0.88	0.02
	0.005	0.0005	1.08	0.92	1.09	1.03	0.10
	0.0005	0.005	0.85	0.93	0.93	0.90	0.05
with BN	0.005	0.005	1.10	1.01	0.93	1.01	0.09
	0.0005	0.0005	0.84	0.88	0.83	0.85	0.03
	0.005	0.0005	1.10	1.12	0.93	1.05	0.10
	0.0005	0.005	0.76	0.82	0.79	**0.79**	0.03

**Table A10:** The performance (percentage error) of **stochastic** classifier with supervised noisy data (noise std = 0.1, # noise realisation = 3) based on $D_{c\hat{c}}+\alpha_{a}D_{a}+\alpha_{c}D_{c}$ for the encoder with and without batch normalization.

Encoder Model	$\alpha_{a}$	$\alpha_{c}$	Runs			Mean	std
			1	2	3		
**MNIST** 100							
without BN	0.005	0.005	12.4	18.05	16.73	15.73	2.96
	0.0005	0.0005	15.01	11.16	14.74	13.64	2.15
	0.005	0.0005	23.31	26.61	25.41	25.11	1.67
	0.0005	0.005	9.21	9.02	10.12	**9.45**	0.59
with BN	0.005	0.005	13.55	22.48	14.72	16.92	4.85
	0.0005	0.0005	8.37	15.01	26.92	16.77	9.40
	0.005	0.0005	32.12	30.27	31.44	31.28	0.94
	0.0005	0.005	5.46	17	11.54	11.33	5.77
**MNIST** 1000							
without BN	0.005	0.005	3.9	4.25	4.02	4.06	0.18
	0.0005	0.0005	3.64	3.82	4.11	3.86	0.24
	0.005	0.0005	6.68	5.34	6.36	6.13	0.70
	0.0005	0.005	3.03	2.88	2.66	2.86	0.19
with BN	0.005	0.005	2.96	3.37	2.98	3.10	0.23
	0.0005	0.0005	2.87	3.10	2.73	2.90	0.19
	0.005	0.0005	3.72	3.8	4.14	3.89	0.22
	0.0005	0.005	2.57	2.39	2.28	**2.41**	0.15
**MNIST** all							
without BN	0.005	0.005	1.05	1.09	1.1	1.08	0.33
	0.0005	0.0005	0.94	0.96	0.9	0.93	0.03
	0.005	0.0005	1.16	1.14	1.13	1.14	0.02
	0.0005	0.005	0.88	0.92	0.91	0.90	0.02
with BN	0.005	0.005	0.98	0.84	0.94	0.92	0.07
	0.0005	0.0005	0.79	0.96	0.82	0.86	0.09
	0.005	0.0005	1.04	1.05	1.03	1.04	0.01
	0.0005	0.005	0.74	0.78	0.84	**0.79**	0.05

## Appendix F. Semi-Supervised Training with Learnable Latent Space Regularization

This model is based on the cross-entropy term $D_{c\hat{c}}$, the MSE term representing $D_{x\hat{x}}$, the label class regularizer $D_{c}$ and either term $D_{z|x}$ or $D_{z}$ or jointly $D_{z|x}$ and $D_{z}$ as defined by (16) in the main part of paper. In our implementation, we consider the regularization of the latent space based on the adversarial term $D_{z}$ only to compare it with the vanila AAE as shown in Figure A7. The encoder is also not conditioned on c as in the original semi-supervised AAE. Thus, the tested system is based on:

(A5) $$L_{\mathrm{SS}-\mathrm{AAE}}^{\mathrm{LP}}\left(\theta_{c},\theta_{x},\phi_{a},\phi_{z}\right)=\beta_{c}D_{c\hat{c}}+\beta_{c}D_{c}+D_{z}+\beta_{x}D_{x\hat{x}}.$$

We set the parameters $\beta_{x}=\beta_{c}=1$ to compare our system with the vanila AAE. However, these parameters can be also optimized in practice.

The parameters of encoder and decoder are shown in Table A11. The performance of this classifier without and with batch normalization is shown in Table A12 (deterministic scenario) and Table A13 (stochastic scenario).

**Table A11:** The encoder and decoder of semi-supervised classifier trained based on $D_{c\hat{c}}$, $D_{c}$ and $D_{z}$. The encoder is trained with and without batch normalization (BN) after Conv2D layers. $D_{c}$ and $D_{z}$ are trained in the adversarial way.

Encoder			
Size		Layer	
28 × 28 × 1 *		Input	
14 × 14 × 32		Conv2D, LeakyReLU	
7 × 7 × 64		Conv2D, LeakyReLU	
4 × 4 × 128		Conv2D, LeakyReLU	
2048		Flatten	
1024		FC, ReLU	
10	10	FC, Softmax	FC
**Decoder**			
Size		Layer	
10 + 10		Input	
7 × 7 × 128		FC, Reshape, BN, ReLU	
14 × 14 × 128		Conv2DTrans, BN, ReLU	
28 × 28 × 128		Conv2DTrans, BN, ReLU	
28 × 28 × 64		Conv2DTrans, BN, ReLU	
28 × 28 × 1		Conv2DTrans, Sigmoid	
**Dz**			
Size		Layer	
10		Input	
500		FC, ReLU	
500		FC, ReLU	
1		FC, Sigmoid	
**Dc**			
Size		Layer	
10		Input	
500		FC, ReLU	
500		FC, ReLU	
1		FC, Sigmoid	

**Table A12:** The performance (percentage error) of **deterministic** classifier based on $D_{c\hat{c}}+D_{c}+D_{z}+D_{x\hat{x}}$ for the encoder with and without batch normalization.

Encoder Model	Runs			Mean	std
	1	2	3		
**MNIST** 100					
without BN	2.15	2.05	1.78	1.99	0.19
with BN	1.57	1.56	1.92	**1.68**	0.21
**MNIST** 1000					
without BN	1.55	1.47	1.53	1.52	0.04
with BN	1.37	1.34	1.73	**1.48**	0.22
**MNIST** all					
without BN	0.78	0.7	0.82	0.77	0.06
with BN	0.79	0.77	0.76	**0.77**	0.02

**Table A13:** The performance (percentage error) of **stochastic** classifier with supervised noisy data (noise std = 0.1, # noise realisation = 3) based on $D_{c\hat{c}}+D_{c}+D_{z}+D_{x\hat{x}}$ for the encoder with and without batch normalization.

Encoder Model	Runs			Mean	std
	1	2	3		
**MNIST** 100					
without BN	1.55	3.19	2.11	2.28	0.83
with BN	1.4	1.33	1.72	**1.48**	0.21
**MNIST** 1000					
without BN	1.73	1.53	1.6	1.62	0.10
with BN	1.28	1.43	1.2	**1.30**	0.12
**MNIST** all					
without BN	0.94	0.86	0.86	0.89	0.05
with BN	0.77	0.65	0.84	**0.75**	0.10

## Appendix G. Semi-Supervised Training with Learnable Latent Space Regularization and Adversarial Reconstruction

This model is similar to the previously considered model but in addition to the MSE reconstruction term representing $D_{x\hat{x}}$ it also contains the adversarial reconstruction term $D_{x}$ as defined by (17) in the main part of paper. In our implementation, we consider the regularization of the latent space based on the adversarial term $D_{z}$ as shown in Figure A8. The training is based on:

(A6) $$L_{\mathrm{SS}-\mathrm{AAE}}^{\mathrm{LP}}\left(\theta_{c},\theta_{x},\phi_{a},\phi_{z}\right)=D_{z}+D_{x\hat{x}}+D_{c\hat{c}}+D_{c}+\alpha_{x}D_{x}.$$

The parameters of encoder and decoder are shown in Table A14. The performance of this classifier without and with batch normalization is shown in Table A15 (deterministic scenario) and Table A16 (stochastic scenario).

**Table A14:** The network parameters of semi-supervised classifier trained based on $D_{c\hat{c}}$, $D_{c}$ and $D_{z}$. The encoder is trained with and without batch normalization (BN) after Conv2D layers. $D_{c}$ and $D_{z}$ are trained in the adversarial way.

Encoder			
Size		Layer	
28 × 28 × 1		Input	
14 × 14 × 32		Conv2D, LeakyReLU	
7 × 7 × 64		Conv2D, LeakyReLU	
4 × 4 × 128		Conv2D, LeakyReLU	
2048		Flatten	
1024		FC, ReLU	
10	10	FC, Softmax	FC
**Dz**			
Size		Layer	
10		Input	
500		FC, ReLU	
500		FC, ReLU	
1		FC, Sigmoid	
**Dc**			
Size		Layer	
10		Input	
500		FC, ReLU	
500		FC, ReLU	
1		FC, Sigmoid	
**Decoder**			
Size		Layer	
10 + 10		Input	
7 × 7 × 128		FC, Reshape, BN, ReLU	
14 × 14 × 128		Conv2DTrans, BN, ReLU	
28 × 28 × 128		Conv2DTrans, BN, ReLU	
28 × 28 × 64		Conv2DTrans, BN, ReLU	
28 × 28 × 1		Conv2DTrans, Sigmoid	
**Dx**			
Size		Layer	
28 × 28 × 1		Input	
14 × 14 × 64		Conv2D, LeakyReLU	
7 × 7 × 64		Conv2D, LeakyReLU	
4 × 4 × 128		Conv2D, LeakyReLU	
4 × 4 × 256		Conv2D, LeakyReLU	
4096		Flatten	
1		FC, Sigmoid	

**Table A15:** The performance (percentage error) of **deterministic** classifier based on $D_{c\hat{c}}+D_{c}+D_{z}+D_{x\hat{x}}+\alpha_{x}D_{x}$ for the encoder with and without batch normalization.

Encoder Model	$\alpha_{x}$	Runs			Mean	std
		1	2	3		
**MNIST** 100						
without BN	0.005	2.85	3.36	2.77	2.99	0.32
	0.0005	2.58	2.49	3.08	2.72	0.32
	1	19.62	19.96	15.97	18.52	2.21
with BN	0.005	1.56	1.33	1.35	**1.41**	0.13
	0.0005	1.68	1.66	2.02	1.79	0.20
	1	20.85	13.6	21.67	18.71	4.44
**MNIST** 1000						
without BN	0.005	2.29	2.35	2.11	2.25	0.12
	0.0005	1.69	1.88	2.24	1.94	0.28
	1	3.47	3.30	4.12	3.63	0.43
with BN	0.005	1.18	1.21	1.09	**1.16**	0.06
	0.0005	1.44	1.28	1.29	1.34	0.09
	1	4.14	2.94	2.48	3.19	0.86
**MNIST** all						
without BN	0.005	0.97	1.01	1.04	1.01	0.04
	0.0005	0.88	0.85	0.93	0.89	0.04
	1	1.31	1.28	1.47	1.35	0.10
with BN	0.005	0.81	0.83	0.75	0.80	0.04
	0.0005	0.73	0.78	0.75	**0.75**	0.03
	1	0.88	0.86	1.27	1.00	0.23

**Table A16:** The performance (percentage error) of **stochastic** classifier with supervised noisy data (noise std = 0.1, # noise realisation = 3) based on $D_{c\hat{c}}+D_{c}+D_{z}+D_{x\hat{x}}+\alpha_{x}D_{x}$ for the encoder with and without batch normalization.

Encoder Model	$\alpha_{x}$	Runs			Mean	std
		1	2	3		
**MNIST** 100						
without BN	0.005	2.45	3.04	2.67	2.72	0.30
	0.0005	2.63	2.3	2.45	2.46	0.17
with BN	0.005	1.34	1.21	6.4	2.98	2.96
	0.0005	1.35	1.51	1.93	**1.60**	0.30
**MNIST** 1000						
without BN	0.005	2.31	2.26	2.2	2.26	0.06
	0.0005	1.71	2.16	1.86	1.91	0.23
with BN	0.005	1.23	1.31	1.10	**1.21**	0.11
	0.0005	1.42	1.62	1.37	1.47	0.13
**MNIST** all						
without BN	0.005	0.93	1.01	1.05	1.00	0.06
	0.0005	0.92	0.83	0.88	0.88	0.05
with BN	0.005	0.88	0.86	0.91	0.88	0.03
	0.0005	0.77	0.80	0.80	**0.79**	0.02
